# Multiomic signatures of body mass index identify heterogeneous health phenotypes and responses to a lifestyle intervention

**DOI:** 10.1038/s41591-023-02248-0

**Published:** 2023-03-20

**Authors:** Kengo Watanabe, Tomasz Wilmanski, Christian Diener, John C. Earls, Anat Zimmer, Briana Lincoln, Jennifer J. Hadlock, Jennifer C. Lovejoy, Sean M. Gibbons, Andrew T. Magis, Leroy Hood, Nathan D. Price, Noa Rappaport

**Affiliations:** 1 Institute for Systems Biology, Seattle, WA USA; 2 Thorne HealthTech, New York, NY USA; 3 Department of Bioengineering, University of Washington, Seattle, WA USA; 4 eScience Institute, University of Washington, Seattle, WA USA; 5 Phenome Health, Seattle, WA USA; 6 Department of Immunology, University of Washington, Seattle, WA USA; 7 Paul G. Allen School of Computer Science and Engineering, University of Washington, Seattle, WA USA; 8 Present Address: Division of Public Health Sciences, Fred Hutchinson Cancer Research Center, Seattle, WA USA

**Keywords:** Computational biology and bioinformatics, Systems biology, Biotechnology, Medical research, Health care

## Abstract

Multiomic profiling can reveal population heterogeneity for both health and disease states. Obesity drives a myriad of metabolic perturbations and is a risk factor for multiple chronic diseases. Here we report an atlas of cross-sectional and longitudinal changes in 1,111 blood analytes associated with variation in body mass index (BMI), as well as multiomic associations with host polygenic risk scores and gut microbiome composition, from a cohort of 1,277 individuals enrolled in a wellness program (Arivale). Machine learning model predictions of BMI from blood multiomics captured heterogeneous phenotypic states of host metabolism and gut microbiome composition better than BMI, which was also validated in an external cohort (TwinsUK). Moreover, longitudinal analyses identified variable BMI trajectories for different omics measures in response to a healthy lifestyle intervention; metabolomics-inferred BMI decreased to a greater extent than actual BMI, whereas proteomics-inferred BMI exhibited greater resistance to change. Our analyses further identified blood analyte–analyte associations that were modified by metabolomics-inferred BMI and partially reversed in individuals with metabolic obesity during the intervention. Taken together, our findings provide a blood atlas of the molecular perturbations associated with changes in obesity status, serving as a resource to quantify metabolic health for predictive and preventive medicine.

## Abstract (web summary)

Integrated analyses reveal that multiomics captured the heterogeneity of metabolic states accompanying obesity and changes in metabolic health in response to lifestyle intervention that are not apparent in body mass index measurements.

## Main

Obesity has been increasing in prevalence over the past four decades in adults, adolescents and children around most of the world^1,2^. Many studies have demonstrated that obesity is a major risk factor for multiple chronic diseases, such as type 2 diabetes mellitus (T2DM), metabolic syndrome (MetS), cardiovascular disease (CVD) and certain types of cancer^3–6^. In individuals with obesity, even a 5% loss in body weight can improve metabolic and cardiovascular health^7^, and weight loss through lifestyle interventions (for example, diet and exercise) can reduce the risk for obesity-related chronic diseases^8^. Nevertheless, obesity and its physiological manifestations can vary widely across individuals, necessitating additional research to better understand this prevalent health condition.

Obesity is commonly quantified using the anthropometric body mass index (BMI), defined as body weight divided by body height squared (kg m^−2^). Although BMI does not directly measure body composition, BMI correlates well at the population level with the body fat percentage measured by specialized devices, such as dual-energy X-ray absorptiometry (DXA)^9^. As an easily calculated and commonly understood measure among researchers, clinicians and the general public, BMI is widely used for the primary diagnosis of obesity, and changes in BMI are often used to assess the effectiveness of lifestyle interventions.

There are limitations to BMI as a surrogate measure of health state. BMI can lead to misclassification of people with a high muscle-to-fat ratio (for example, athletes) as individuals with obesity and can undervalue metabolic improvements in health after exercise^10^. A meta-analysis showed that the common obesity diagnoses based on BMI cutoffs had high specificity but low sensitivity in identifying individuals with excess body fat^11^. The misclassification is likely due, in part, to the differences in BMI thresholds for obesity across different ethnic populations^12^ as well as the existence of a metabolically unhealthy, normal-weight (MUNW) group within the normal BMI class^13,14^. Likewise, there are health-heterogeneous groups among individuals with obesity: metabolically healthy obese (MHO) and metabolically unhealthy obese (MUO). Although most individuals in the MHO group are not necessarily healthy but simply healthier than individuals in the MUO group^15^, the transition from MHO to MUO phenotype may be a preceding step to the development of obesity-related chronic diseases^16^. Moreover, this transition is potentially preventable through lifestyle interventions^17^. Hence, BMI is unequivocally useful at the population level but too crude to capture a variety of heterogeneous metabolic health states.

Omics studies have demonstrated how blood omic profiles contain information relevant to a wide range of human health conditions; for example, blood proteomics captured 11 health indicators, such as the liver fat measured by ultrasound and the body composition measured by DXA^18^, whereas blood metabolomics tended to reflect dietary intake, lifestyle patterns and gut microbiome profiles^19,20^. A machine learning model that was trained to predict BMI using 49 BMI-associated blood metabolites captured obesity-related clinical measurements (for example, visceral fat percentage) better than observed BMI or genetic predisposition for high BMI^21^. Moreover, another blood metabolomics-based model of BMI reflected differences between individuals with or without acute coronary syndrome^22^. Thus, although a single targeted metric (for example, body composition) or a specific biomarker (for example, leptin^23^) provides useful information, multiomic blood profiling has the potential to comprehensively bridge the multifaceted gaps between BMI and heterogeneous physiological states.

Here we report heterogeneous molecular signatures of obesity by leveraging a cohort of 1,277 individuals with phenotype data, including human genomes and longitudinal measurements of metabolomics, proteomics, clinical laboratory tests, gut microbiomes, physical activity (that is, wearables) and health/lifestyle questionnaires, and by employing machine learning to predict BMI.

## Results

### Arivale cohort characteristics

**Fig. 1:**
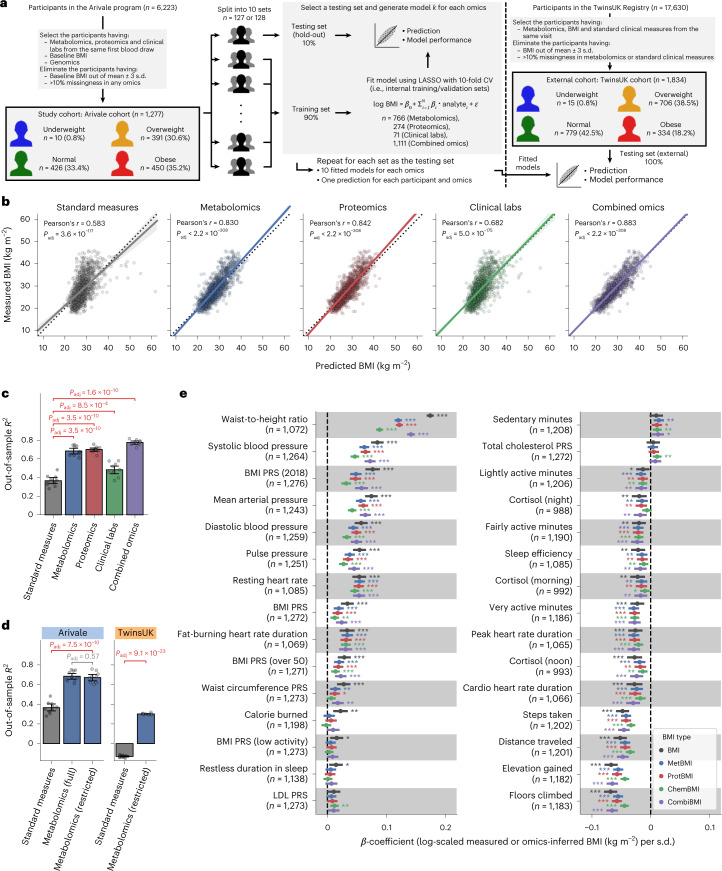
Plasma multiomics captured 48–78% of the variance in BMI. **a**, Overview of study cohorts and the omics-based BMI model generation. CV, cross-validation. **b**, Correlation between the measured and predicted BMIs. The solid line is the OLS linear regression line with 95% confidence interval, and the dotted line is measured BMI = predicted BMI. Standard measures: OLS linear regression model with sex, age, triglycerides, HDL cholesterol, LDL cholesterol, glucose, insulin and HOMA-IR as regressors; *P*_adj_: adjusted *P* value of two-sided Pearson’s correlation test with the Benjamini–Hochberg method across the five categories (*n* = 1,277 participants). **c**,**d**, Model performance of each fitted BMI model. Out-of-sample *R*^2^ was calculated from each corresponding hold-out testing set (Arivale: **c**,**d**) or from the external testing set (TwinsUK: **d**). Metabolomics (full): LASSO model trained by all 766 metabolites of the Arivale dataset; Metabolomics (restricted): LASSO model trained by the common 489 metabolites in the Arivale and TwinsUK datasets (Extended Data Fig. 3 and Methods); *P*_adj_: adjusted *P* value of two-sided Welch’s *t*-test with the Benjamini–Hochberg method across the four (**c**) or three (**d**) comparisons. Data: mean with 95% confidence interval, *n* = 10 models. Note that Standard measures and Metabolomics (full) of Arivale in **d** are the same with corresponding ones in **c**. **e**, Association between omics-inferred BMI and physiological feature. For each of the 51 numeric physiological features (Supplementary Data 4), *β*-coefficient was estimated using OLS linear regression model with the measured or omics-inferred BMI as a dependent variable and sex, age and ancestry principal components as covariates. Presented are the 30 features that were significantly associated with at least one of the BMI types after multiple testing adjustment with the Benjamini–Hochberg method across the 255 (51 features × 5 BMI types) regressions. *n,* number of assessed participants. Data: estimate with 95% confidence interval. *Adjusted *P* < 0.05, **adjusted *P* < 0.01, ***adjusted *P* < 0.001. All exact values of test summaries are found in Supplementary Data 4 and 10.

**Extended Data Fig. 1:**
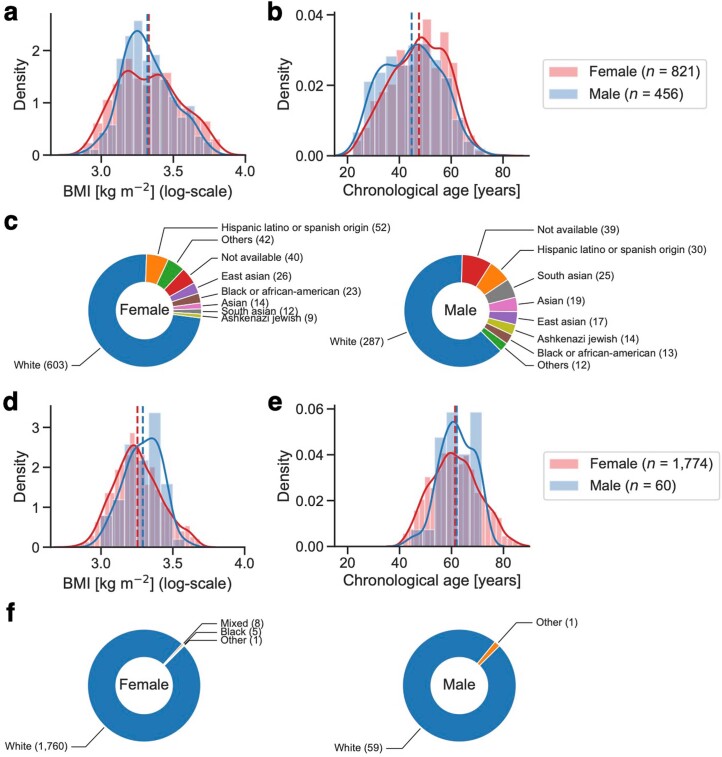
Demographic information of study cohorts. **a**–**c**, Demographic information of the Arivale study cohort (Fig. 1a, *n* = 1,277 participants). **d**–**f**, Demographic information of the TwinsUK study cohort (Fig. 1a, *n* = 1,834 participants). **a**,**b**,**d**,**e**, Distribution of the baseline BMI (**a**,**d**) or age (**b**,**e**). *n*, number of participants. The solid and dashed lines indicate the kernel density estimate and the mean of BMI (**a**, Female: 28.6 kg m^−2^; **a**, Male: 28.1 kg m^−2^; **d**, Female: 26.2 kg m^−2^; **d**, Male: 27.1 kg m^−2^) or age (**b**, Female: 47.6 years; **b**, Male: 44.7 years; **e**, Female: 61.4 years; **e**, Male: 62.0 years), respectively. **c**,**f**, Composition of self-reported race (**c**) or ethnicity (**f**). The number in parentheses indicates the number of participants. All summary values are found in Supplementary Data 1.

We selected a study cohort of 1,277 adults who participated in a scientific wellness program (Arivale)^20,24–29^ and had coupled measurements of plasma metabolomics, proteomics and clinical laboratory tests from the same blood draw (Fig. 1a and Methods). This study design allowed us to directly investigate the similarities and differences between omics platforms according to the physiological health state of each individual across the BMI spectrum. This cohort was characteristically female (64.3%), middle-aged (mean ± s.d.: 46.6 ± 10.8 years) and White (69.7%) (Extended Data Fig. 1a–c and Supplementary Data 1). Based on the World Health Organization (WHO) international standards for BMI cutoffs (underweight: <18.5 kg m^−2^, normal: 18.5–25 kg m^−2^, overweight: 25–30 kg m^−2^, obese: ≥30 kg m^−2^)^12^, the baseline BMI prevalence was similar among normal, overweight and obese classes, whereas only 0.8% of participants were in the underweight class (underweight: ten participants (0.8%), normal: 426 participants (33.4%), overweight: 391 participants (30.6%), obese: 450 participants (35.2%)). In the Arivale program, personalized healthy lifestyle coaching was provided to all participants (Methods), resulting in clinical improvement across multiple measures of health^25^.

### Blood omics-based BMI models

**Extended Data Fig. 2:**
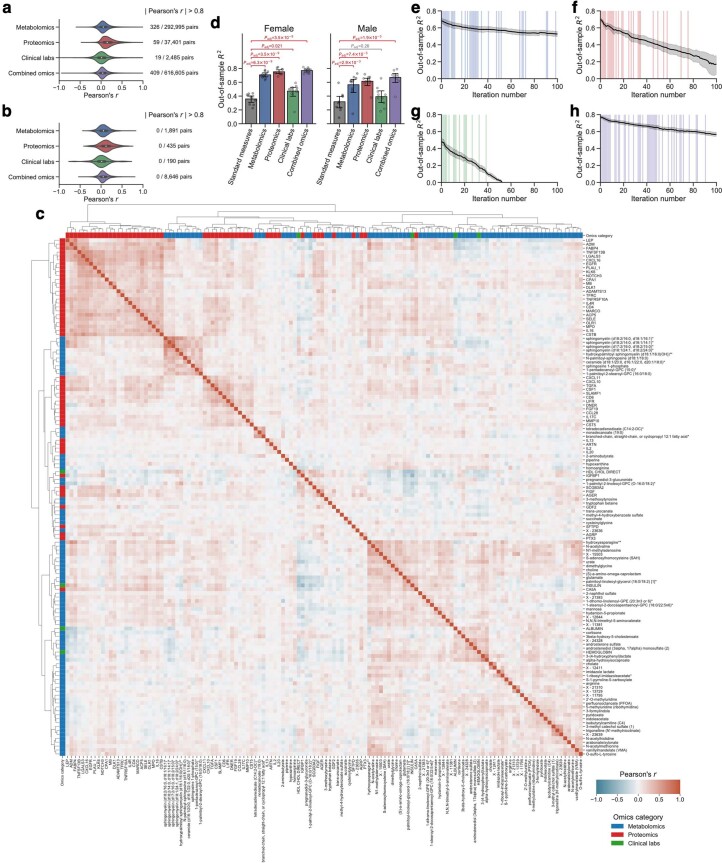
Quality check of the LASSO modeling. **a**,**b**, Pairwise correlation of all plasma analytes (**a**; Metabolomics: 766 metabolites, Proteomics: 274 proteins, Clinical labs: 71 clinical laboratory tests, Combined omics: 1,111 analytes) or the analytes that were retained across all ten LASSO models (**b**; Metabolomics: 62 metabolites, Proteomics: 30 proteins, Clinical labs: 20 clinical laboratory tests, Combined omics: 132 analytes). Each violin is scaled to have same width between the omics categories and represents the kernel density distribution with the standard boxplot (Methods). **c**, Hierarchical clustering and heatmap for the pairwise correlations of the analytes that were retained across all ten CombiBMI models (132 analytes: 77 metabolites, 51 proteins and four clinical laboratory tests). Of note, both upper and lower triangular sides of the symmetric matrix are visualized. **d**, Model performance of each fitted BMI model with sex stratification. Out-of-sample *R*^2^ was calculated from each corresponding hold-out testing set. Standard measures: OLS linear regression model with sex, age, triglycerides, HDL cholesterol, LDL cholesterol, glucose, insulin and HOMA-IR as regressors; *P*_adj_: adjusted *P* value of two-sided Welch’s *t*-test with the Benjamini–Hochberg method across the eight (four comparisons × two sexes) comparisons. Data: mean with 95% confidence interval, *n* = 10 models. All exact values of test summaries are found in Supplementary Data 10. Note that the sample size for modeling was different between female and male (Female: 821 participants versus Male: 456 participants). **e**–**h**, Transition of out-of-sample *R*^2^ in the LASSO-modeling iteration analysis (Methods) for metabolomics (**e**), proteomics (**f**), clinical labs (**g**) or combined omics (**h**). The iteration is highlighted with shading color when the removed analyte is the variable that was retained across all the original ten models. Data: mean with 95% confidence interval, *n* = 10 models.

Leveraging the baseline measurements of plasma molecular analytes (766 metabolites, 274 proteins and 71 clinical laboratory tests; Supplementary Data 2), we trained machine learning models to predict baseline BMI for each of the omics platforms (metabolomics, proteomics and clinical labs) or in combination: metabolomics-based BMI (MetBMI), proteomics-based BMI (ProtBMI), clinical labs (chemistries)-based BMI (ChemBMI) and combined omics-based BMI (CombiBMI) models. To address multicollinearity among the analytes (Extended Data Fig. 2a) and to obtain predictions for all participants, we applied a ten-fold iteration scheme of the least absolute shrinkage and selection operator (LASSO) algorithm with ten-fold cross-validation (Fig. 1a and Methods). This approach generated ten fitted sparse models for each omics category (Supplementary Data 3) and one single testing (hold-out) set-derived prediction from each omics category for each participant (Fig. 1b). The resulting models retained 62 metabolites, 30 proteins, 20 clinical laboratory tests and 132 analytes across all ten MetBMI, ProtBMI, ChemBMI and CombiBMI models, respectively, which exhibited low collinearity (Extended Data Fig. 2b,c) as expected from the LASSO algorithm^30^. In contrast to a model including obesity-related standard clinical measures (that is, ordinary least squares (OLS) linear regression model with sex, age, triglycerides, high-density lipoprotein (HDL) cholesterol, low-density lipoprotein (LDL) cholesterol, glucose, insulin and homeostatic model assessment for insulin resistance (HOMA-IR) as regressors; StandBMI model), each omics-based model demonstrated significantly higher performance in BMI prediction, ranging from out-of-sample *R*^2^ = 0.48 (ChemBMI) to 0.70 (ProtBMI) compared to 0.37 (StandBMI) (Fig. 1c). The CombiBMI model exhibited the best performance in BMI prediction (out-of-sample *R*^2^ = 0.78; Fig. 1c), but the variances explained were not completely additive, suggesting that, although there is a considerable overlap in the signal detected by each omics platform, different omic measurements still contain non-redundant information regarding BMI. Additionally, these results were consistent in sex-stratified models, with the exception of the male ChemBMI model that exhibited higher performance than the StandBMI model without statistical significance (Extended Data Fig. 2d).

**Extended Data Fig. 3:**
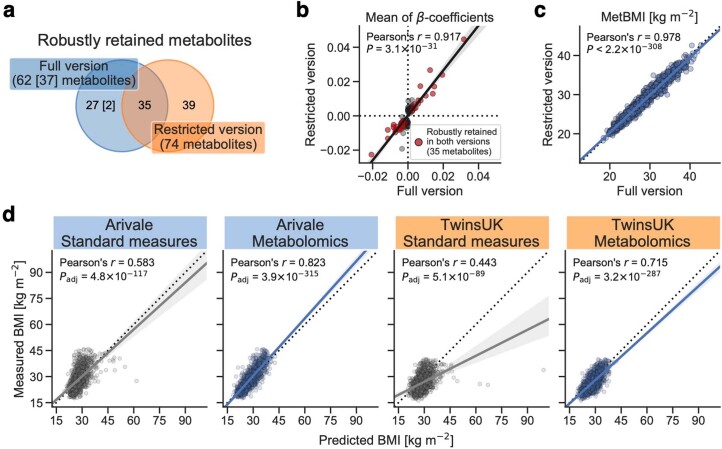
The restricted MetBMI model predominantly maintained the characteristics of the original full model. **a**–**c**, Comparison of the MetBMI model between the main analyses (Arivale cohort) and the validation analyses (TwinsUK cohort). Full version: LASSO model trained by all 766 metabolites in the Arivale dataset, Restricted version: LASSO model trained by the common 489 metabolites in the Arivale and TwinsUK datasets (Methods). **a**, The number of the variables that were robustly retained across all ten MetBMI models. The number in square brackets indicates the number of the robustly retained metabolites that were derived from the common 489 metabolites. **b**, Correlation of the mean of *β*-coefficients in the ten MetBMI models. Only the robustly retained metabolites in either full version (37 metabolites) or restricted version (74 metabolites) were analyzed. **c**, Correlation of the MetBMI prediction. **b**,**c**, The solid line is the OLS linear regression line with 95% confidence interval, and the dotted line in **c** is the value in full version = the value in restricted version. *P*: *P* value of two-sided Pearson’s correlation test. *n* = 76 metabolites (**b**) or 1,277 participants (**c**). **d**, Correlation between the measured and predicted BMIs. The solid line is the OLS linear regression line with 95% confidence interval, and the dotted line is measured BMI = predicted BMI. Standard measures: OLS linear regression model with sex, age, triglycerides, HDL cholesterol, LDL cholesterol, glucose, insulin and HOMA-IR as regressors; Metabolomics: the restricted version of MetBMI model, corresponding to Metabolomics (restricted) in Fig. 1d; *P*_adj_: adjusted *P* value of two-sided Pearson’s correlation test with the Benjamini–Hochberg method across the four (two categories × two cohorts) tests. *n* = 1,277 (Arivale) or 1,834 (TwinsUK) participants. All exact values of test summaries are found in Supplementary Data 10.

To confirm the generalizability of our results, we investigated an external cohort of 1,834 adults from the TwinsUK registry^31^ whose datasets included serum metabolomics^32^ and the aforementioned standard clinical measures (Fig. 1a, Extended Data Fig. 1d–f and Supplementary Data 1). We calculated BMI predictions for the TwinsUK cohort using the StandBMI and restricted MetBMI models that were fitted to the Arivale datasets (Extended Data Fig. 3 and Methods). The restricted MetBMI model exhibited a lower absolute performance on the TwinsUK cohort compared to the Arivale cohort but a significantly higher performance than the StandBMI model (out-of-sample *R*^2^ = 0.30 (MetBMI) and −0.13 (StandBMI); Fig. 1d), confirming that blood metabolomics generally captures BMI better than the standard clinical measures.

BMI has been reported to be associated with multiple anthropometric and clinical measures, such as waist circumference, blood pressure, sleep quality and several polygenic risk scores (PRSs)^3,4,15,27,33^. We examined the association between the omics-inferred BMI and each of the available numeric physiological measures (Methods and Supplementary Data 4). Among the 51 assessed features, classically measured BMI was significantly associated with 27 features (false discovery rate (FDR) < 0.05), including daily physical activity measures from wearable devices, waist-to-height ratio (WHtR), blood pressure and BMI PRS (Fig. 1e). With minor differences in effect sizes, these BMI-associated features were concordantly associated with each omics-inferred BMI (Fig. 1e), indicating that the omics-inferred BMIs primarily maintain the characteristics of classical BMI in terms of anthropometric, genetic, lifestyle and physiological associations.

### Predictive features in omics-based BMI models

**Fig. 2:**
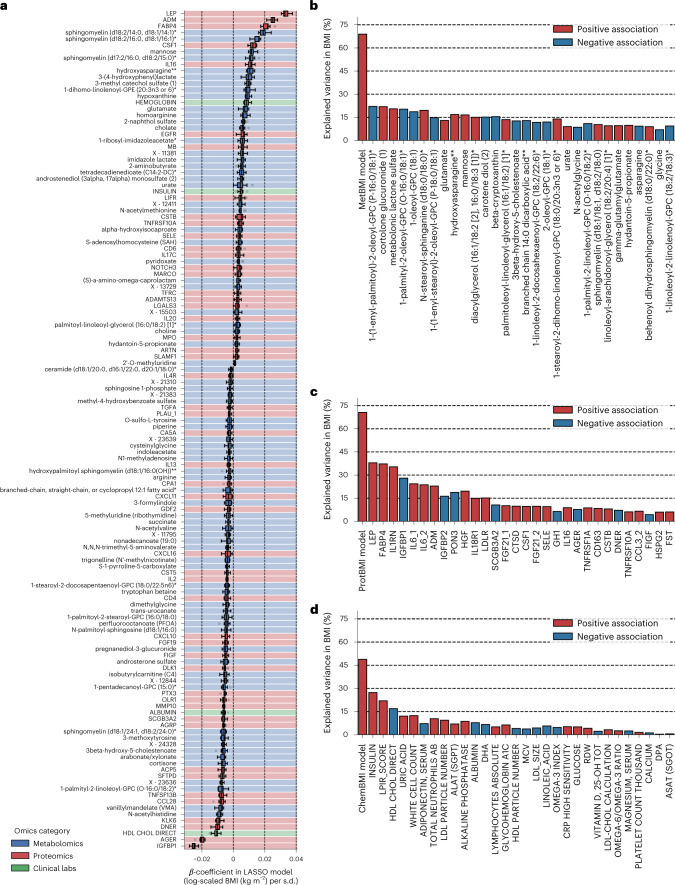
Omics-based BMI estimates captured the variance in BMI better than any single analyte. **a**, The variables that were retained across all ten CombiBMI models (132 analytes: 77 metabolites, 51 proteins and four clinical laboratory tests). *β*-coefficient was obtained from the fitted CombiBMI model with LASSO linear regression (Supplementary Data 3). Each background color corresponds to the analyte category. Data: the standard box plot (Methods), *n* = 10 models. **b**–**d**, Univariate explained variance in BMI by each metabolite (**b**), protein (**c**) or clinical laboratory test (**d**). BMI was independently regressed on each of the analytes that were retained in at least one of the ten LASSO models (209 metabolites, 74 proteins and 41 clinical laboratory tests; Supplementary Data 5), using OLS linear regression with sex, age and ancestry principal components as covariates. Multiple testing was adjusted with the Benjamini–Hochberg method across the 210 (**b**), 75 (**c**) or 42 (**d**) regressions, including each omics-based BMI model as reference. Among the analytes that were significantly associated with BMI (180 metabolites, 63 proteins and 30 clinical laboratory tests), only the top 30 significant analytes are presented with their univariate variances. All exact values of test summaries are found in Supplementary Data 5.

**Extended Data Fig. 4:**
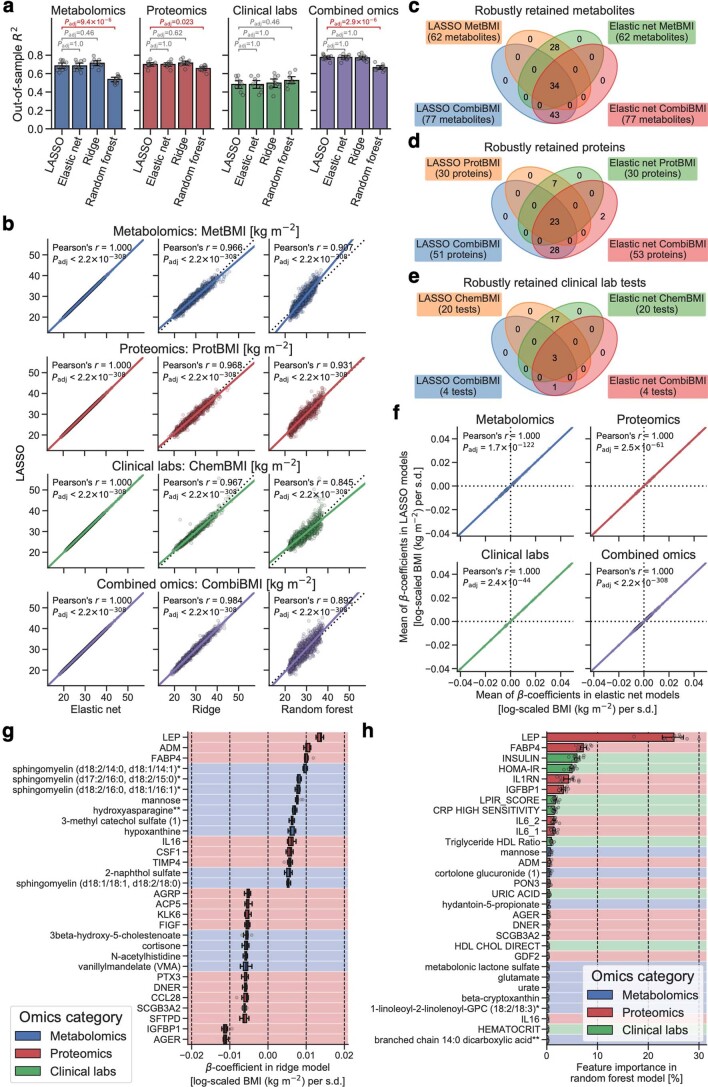
Omics-based BMI models were similar between LASSO and the other methods. **a**, Model performance of each fitted BMI model. *P*_adj_: adjusted *P* value of two-sided Welch’s *t*-test with the Benjamini–Hochberg method across the 12 (3 methods × 4 categories) comparisons. Data: mean with 95% confidence interval, *n* = 10 models. **b**, Correlation of the predicted BMI between LASSO and the other methods. The solid line is the OLS linear regression line with 95% confidence interval, and the dotted line is LASSO = the other method. *P*_adj_: adjusted *P* value of two-sided Pearson’s correlation test with the Benjamini–Hochberg method across the 12 (3 methods × 4 categories) combinations. *n* = 1,277 participants. **c**–**f**, Comparison of the omics-based BMI model between LASSO and elastic net. **c**–**e**, The number of the variables that were robustly retained across all ten models. **f**, Correlation of the mean of *β*-coefficients in the ten models. Only the robustly retained analytes in either LASSO models or elastic net models were analyzed. The solid line is the OLS linear regression line with 95% confidence interval. *P*_adj_: adjusted *P* value of two-sided Pearson’s correlation test with the Benjamini–Hochberg method across the four categories. *n* = 62 metabolites, 30 proteins, 20 clinical laboratory tests or 134 analytes. **a**,**b**,**f**, All exact values of test summaries are found in Supplementary Data 10. **g**, The top 30 variables that had the highest absolute value for the mean of *β*-coefficients in the ten ridge CombiBMI models. *β*-coefficient was obtained from the fitted CombiBMI model with ridge linear regression. Data: the standard box plot (Methods), *n* = 10 models. **h**, The top 30 variables that had the highest mean of feature importance in the ten random forest CombiBMI models. Feature importance was calculated as the normalized total reduction of the mean squared error. Data: mean with 95% confidence interval, *n* = 10 models.

**Extended Data Fig. 5:**
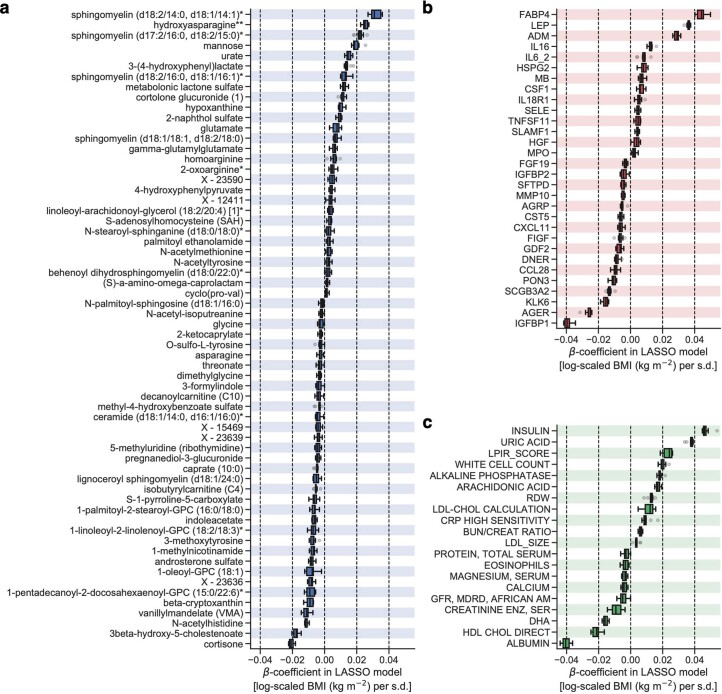
Variable diversity and contribution to the omics-based BMI model were different between omics categories. **a**–**c**, The variables that were retained across all ten MetBMI (**a**), ProtBMI (**b**) or ChemBMI (**c**) models (**a**: 62 metabolites, **b**: 30 proteins, **c**: 20 clinical laboratory tests). *β*-coefficient was obtained from the fitted omics-based BMI model with LASSO linear regression (Supplementary Data 3). Data: the standard boxplot (Methods), *n* = 10 models.

Because our LASSO linear regression model showed similar performance to elastic net and ridge linear regression models and a non-linear random forest regression model (Extended Data Fig. 4a,b), and because the LASSO model’s *β*-coefficients are generally easier to interpret, we chose to focus on the LASSO models. However, the LASSO algorithm randomly retains variables from highly collinear groups and sets *β*-coefficients of the other variables to 0. To confirm the robustness of the variable selection process, we iterated the LASSO modeling while removing the strongest analyte (that is, the analyte that had the highest absolute value for the mean of the ten *β*-coefficients) from the input omic dataset at the end of each iteration. If a variable is indispensable for a model, the performance should largely decrease after removing it. In all omics categories, a steep decay in the out-of-sample *R*^2^ was observed in the first 5–9 iterations (Extended Data Fig. 2e–h), suggesting that, at least, the 5–9 analytes that had the highest absolute *β*-coefficients in the original LASSO models were indispensable for predicting BMI. Compared to ProtBMI and ChemBMI models, the overall slope of *R*^2^ in the MetBMI model decayed more gradually (Extended Data Fig. 2e–g), and the proportion of the variables that were robustly retained across all ten LASSO models (Extended Data Fig. 5) to the variables that were retained in at least one of the ten LASSO models was lower in the MetBMI model (MetBMI: 62/209 metabolites ≈30%; ProtBMI: 30/74 proteins ≈41%; ChemBMI: 20/41 clinical laboratory tests ≈49%), implying that metabolomics data contain more redundant information about BMI than the other omics data. Nevertheless, metabolites still constituted 58% of the 132 analytes that were retained across all ten CombiBMI models (77 metabolites, 51 proteins and four clinical laboratory tests; Fig. 2a), suggesting that each of the omics categories possesses unique information about BMI. The strongest predictors in the CombiBMI model were primarily proteins; analytes having the mean absolute *β*-coefficient >0.02 were leptin (LEP), adrenomedullin (ADM) and fatty acid-binding protein 4 (FABP4) as the positive predictors and insulin-like growth factor-binding protein 1 (IGFBP1) and advanced glycosylation end-product-specific receptor (AGER; also called RAGE) as the negative predictors. These strongest proteins were consistent in the elastic net models (Extended Data Fig. 4c–f) and had high importance in the ridge and random forest models (Extended Data Fig. 4g,h).

These consistently retained predictors in the omics-based BMI models implied that a single analyte might be a suitable biomarker to predict BMI. To address this possibility, we assessed the association between each single analyte and BMI for the analytes that were retained in at least one of the ten LASSO models (MetBMI: 209 metabolites, ProtBMI: 74 proteins and ChemBMI: 41 clinical laboratory tests; Supplementary Data 5). Among the analytes that were significantly associated with BMI (180 metabolites, 63 proteins and 30 clinical laboratory tests), only LEP, FABP4 and interleukin 1 receptor antagonist (IL1RN) exhibited over 30% of the explained variance in BMI by themselves (Fig. 2b–d), with a maximum of 37.9% variance explained (LEP). In contrast, MetBMI, ProtBMI and ChemBMI models explained 68.9%, 70.6% and 48.8% of the variance, respectively. Moreover, even upon eliminating several strong analytes (for example, LEP and FABP4) from the omic datasets, the models still explained more variance in BMI than any single analyte (Extended Data Fig. 2e–h). These results indicate that the multiomic BMI prediction models explain a larger portion of the variation in BMI than any single analyte and highlight the multivariable perturbation of blood analytes across all platforms with increasing BMI.

### Metabolic heterogeneity within the standard BMI classes

**Fig. 3:**
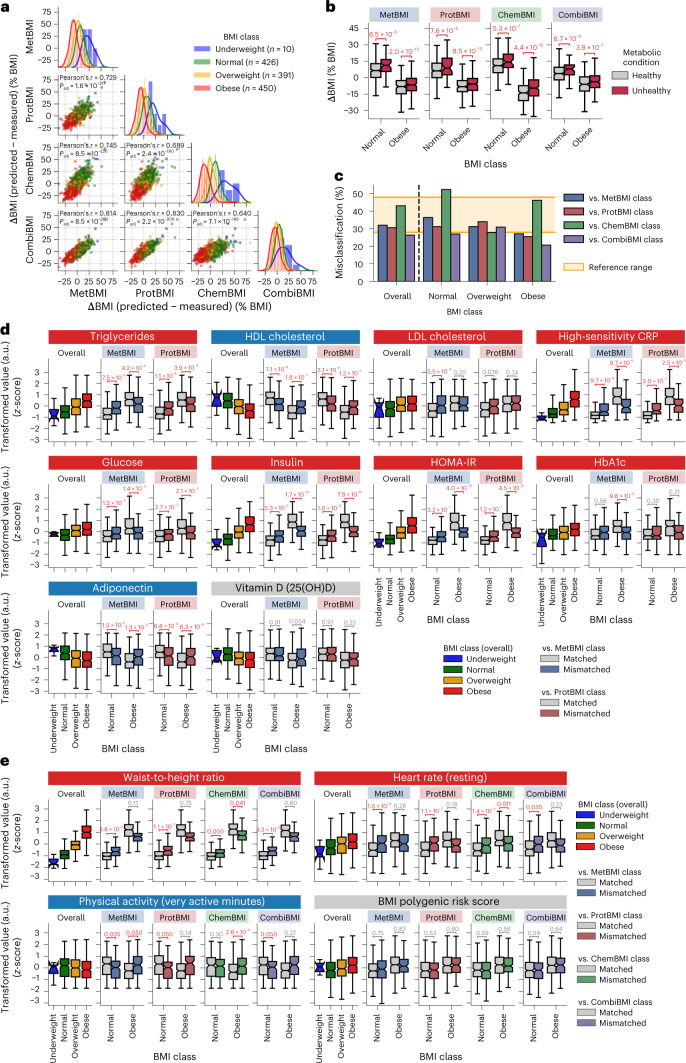
Metabolic heterogeneity was responsible for the high rate of misclassification within the standard BMI classes. **a**, Difference of the omics-inferred BMI from the measured BMI (ΔBMI). *P*_adj_: adjusted *P* value of two-sided Pearson’s correlation test with the Benjamini–Hochberg method across the six combinations (*n*, number of participants in each BMI class; total *n* = 1,277 participants). The line in the histogram panel indicates the kernel density estimate. **b**, Difference in ΔBMI between clinically defined metabolic health conditions within the normal or obese BMI class. Each comparison value indicates adjusted *P* value, calculated from OLS linear regression with BMI, sex, age and ancestry principal components as covariates while adjusting multiple testing with the Benjamini–Hochberg method across the eight (2 BMI classes × 4 omics categories) regressions. **c**, Misclassification rate of overall cohort or each BMI class against the omics-inferred BMI class. Reference range: the previously reported misclassification rate^36,37^. The underweight BMI class is not presented owing to small sample size, but its misclassification rate was 80% against CombiBMI class and 100% against the others. **d**,**e**, Difference in the obesity-related clinical blood marker (**d**) or BMI-associated physiological feature (**e**) between the matched and mismatched groups within the normal or obese BMI class. Each comparison value indicates adjusted *P* value, calculated from OLS linear regression with BMI, sex, age and ancestry principal components as covariates while adjusting multiple testing with the Benjamini–Hochberg method across the 40 (**d**, 2 BMI classes × 2 omics categories × 10 markers) or 216 (**e**, 2 BMI classes × 4 omics categories × 27 features) regressions. Four of the 27 features that were significantly associated with BMI (Fig. 1c) are representatively presented in **e**, and the other results are found in Supplementary Data 6. 25(OH)D, 25-hydroxyvitamin D; a.u., arbitrary units. **b**,**d**,**e**, Data: the standard box plot (Methods); *n* = 373 (**b**, Healthy in Normal), 49 (**b**, Unhealthy in Normal), 208 (**b**, Healthy in Obese) or 241 (**b**, Unhealthy in Obese) participants (see Supplementary Data 6 for each sample size in **d** and **e**). All exact values of test summaries are found in Supplementary Data 6 and 10.

Although the omics-inferred BMIs showed the similar phenotypic associations as classical BMI (Fig. 1e), we observed that the difference of the predicted BMI from the measured BMI (ΔBMI) was highly correlated among the omics categories, ranging from Pearson’s *r* = 0.64 (ChemBMI versus CombiBMI) to 0.83 (ProtBMI versus CombiBMI) (Fig. 3a), implying that this deviation stemmed from a true biological signal of a perturbed physiological state rather than from noise or modeling artifacts. When individuals in the normal and obese BMI classes (defined by the WHO international standards) were subdivided by a clinical definition of metabolic health (that is, defining metabolically unhealthy if having two or more MetS risks; Methods)^34,35^, ΔBMI was significantly higher in MUNW and MUO groups compared to metabolically healthy, normal-weight (MHNW) and MHO groups, respectively, for all omics categories (Fig. 3b), suggesting that the deviations of model predictions are related to metabolic health.

Nevertheless, there has been no universally accepted definition of metabolic health^14,15,34,35^. Given the high interpretability and intuitiveness of the omics-inferred BMI, we explored a potential application: using the omics-inferred BMI (instead of actual BMI) for improved classification of both obesity and metabolic health with the WHO international standards. Each participant was classified using each of the measured and omics-inferred BMIs based on the standard BMI cutoffs and categorized into either a matched or a mismatched group when the measured BMI class was matched or mismatched to each omics-inferred BMI class, respectively. The misclassification rate against the omics-inferred BMI class was ~30% across all omics categories and BMI classes (Fig. 3c), consistent with the previously reported misclassification rates about the cardiometabolic health classification^36,37^. We then examined relationships between this omics-based misclassification within normal or obese BMI class and the obesity-related clinical blood markers (Supplementary Data 6), including triglycerides, HDL cholesterol, LDL cholesterol, high-sensitivity C-reactive protein (hs-CRP), glucose, insulin, HOMA-IR, glycated hemoglobin A1c (HbA1c), adiponectin and vitamin D^3,15,23,38,39^. Because ChemBMI and CombiBMI models were not independent of these markers, only the misclassification against MetBMI or ProtBMI class was examined in this analysis. The mismatched group of normal BMI class exhibited significantly higher values of the markers that are positively associated with BMI (+_BMI_), including triglycerides, hs-CRP, glucose and HOMA-IR, and significantly lower values of the markers that are negatively associated with BMI (−_BMI_), including HDL cholesterol and adiponectin, compared to the matched group of normal BMI class (FDR < 0.05; Fig. 3d). These patterns suggest that the participant misclassified into the normal BMI class possesses less healthy molecular profiles comparable to an individual with overweight or obesity, corresponding to the individual with MUNW phenotype. Conversely, the mismatched group of obese BMI class exhibited significantly lower and higher values of the positively and negatively BMI-associated markers, respectively, compared to the matched group of obese BMI class (FDR < 0.05; Fig. 3d), suggesting that the participant misclassified as obese BMI class has healthier blood signatures comparable to an individual with overweight or normal weight, corresponding to the individual with MHO phenotype.

**Extended Data Fig. 6:**
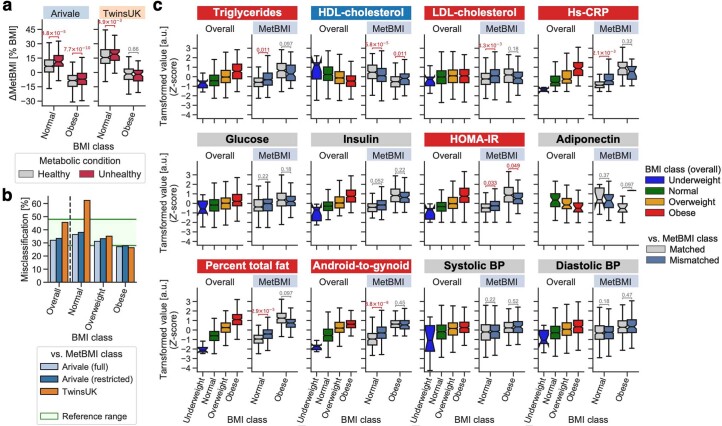
The metabolic heterogeneity within the standard BMI classes was validated with the TwinsUK cohort. **a**, Difference in ΔMetBMI (that is, difference of MetBMI from the measured BMI) between clinically-defined metabolic health conditions. Each comparison value indicates adjusted *P* value, calculated from OLS linear regression with BMI, sex and age as covariates, while adjusting multiple testing with the Benjamini–Hochberg method across the four (two BMI classes × two cohorts) regressions. For Arivale cohort, ancestry principal components were also included in the covariates. MetBMI in Arivale was derived from the restricted version of MetBMI model (Extended Data Fig. 3 and Methods). **b**, Misclassification rate of overall cohort or each BMI class against MetBMI class. Arivale (full): based on the full version of MetBMI model in Extended Data Fig. 3 (that is, the same with the corresponding ones in Fig. 3c), Arivale (restricted): based on the restricted version of MetBMI model in Extended Data Fig. 3, Reference range: the previously reported misclassification rate^36,37^. The underweight BMI class is not presented owing to small sample size, but its misclassification rate was 100% in both cohorts. **c**, Difference in the obesity-related phenotypic measure between the matched and mismatched groups in the TwinsUK cohort. Each comparison value indicates adjusted *P* value, calculated from OLS linear regression with BMI, sex and age as covariates, while adjusting multiple testing with the Benjamini–Hochberg method across the 24 (2 BMI classes × 12 measures) regressions. Percent total fat: percentage of total fat in the whole body, Android-to-gynoid: ratio of fat in the android region to fat in the gynoid region, BP: blood pressure, a.u.: arbitrary units. **a**,**c**, Data: the standard boxplot (Methods). See Supplementary Data 6 for the number of participants in each group. All exact values of test summaries are found in Supplementary Data 6 and 10.

We re-examined the 27 BMI-associated numeric physiological features (Fig. 1e and Supplementary Data 6) as well and found the concordant pattern of significant phenotypic differences between the matched and mismatched groups in WHtR (+_BMI_), heart rate (+_BMI_), blood pressure (+_BMI_) and daily physical activity (−_BMI_) measures (FDR < 0.05; Fig. 3e). There was no difference in BMI PRS (+_BMI_) between the matched and mismatched groups (Fig. 3e), implying that lifestyle or environmental factors, rather than genetic risk, are likely associated with the discordance between the measured and omics-inferred BMIs. Furthermore, we validated these findings using the TwinsUK cohort (Extended Data Fig. 6). Taken together, these results suggest that the omics-inferred BMIs are associated with heterogeneous metabolic health states that are not captured by classical BMI with the standard BMI cutoffs.

### Abdominal obesity and omics-based BMI models

**Extended Data Fig. 7:**
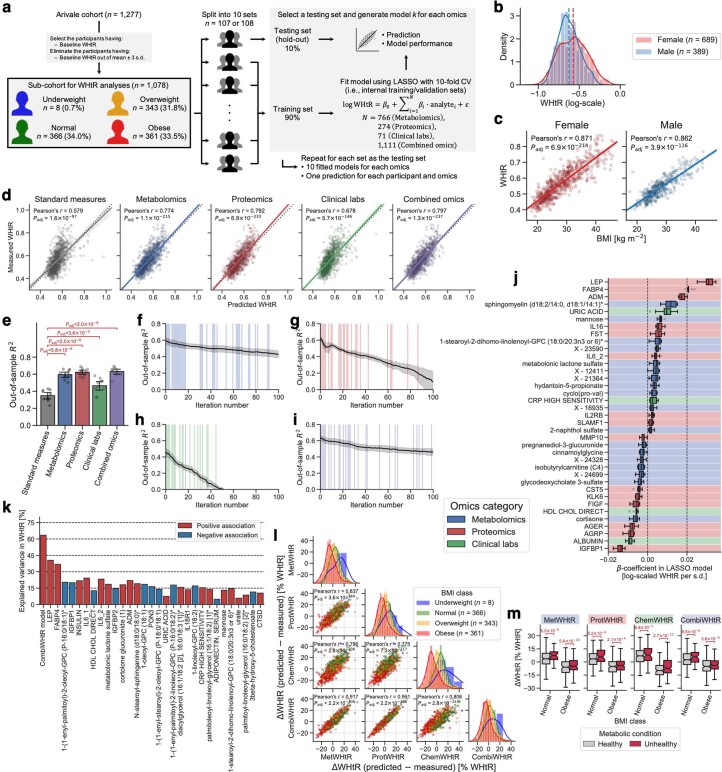
Omics-based WHtR models consistently supported the findings of omics-based BMI models. **a**, Overview of study cohort and the omics-based WHtR model generation. CV,cross-validation. **b**, Distribution of the baseline WHtR. **c**, Correlation between the measured WHtR and BMI. **d**, Correlation between the measured and predicted WHtRs. **e**, Model performance of each fitted WHtR model. **f**–**i**, Transition of out-of-sample *R*^2^ in the LASSO-modeling iteration analysis (Methods) for metabolomics (**f**), proteomics (**g**), clinical labs (**h**) or combined omics (**i**). The iteration is highlighted with shading color when the removed analyte is the variable that was retained across all the original ten models. **j**, The variables that were retained across all ten CombiWHtR models (37 analytes: 18 metabolites, 15 proteins and four clinical laboratory tests). *β*-coefficient was obtained from the fitted CombiWHtR model (Supplementary Data 8). **k**, Univariate explained variance in WHtR by each analyte. Among the analytes that were significantly associated with WHtR (212 analytes; Methods), only the top 30 significant analytes are presented with their univariate variances. **l**, Difference of the omics-inferred WHtR from the measured WHtR (ΔWHtR). **m**, Difference in ΔWHtR between clinically-defined metabolic health conditions. Each comparison value indicates adjusted *P* value, calculated from OLS linear regression with WHtR, sex, age and ancestry principal components as covariates, while adjusting multiple testing with the Benjamini–Hochberg method across the eight (two BMI classes × four omics categories) regressions. *P*_adj_: adjusted *P* value of two-sided Pearson’s correlation test (**c**,**d**,**l**) or Welch’s *t*-test (**e**) with the Benjamini–Hochberg method across the two sexes (**c**), five categories (**d**), four comparisons (**e**) or six combinations (**l**). Data: mean with 95% confidence interval (**e**–**i**) or the standard boxplot (**j**,**m**), *n* = 10 models (**e**–**i**,**j**) (see Supplementary Data 10 for each number of participants in **m**). All exact values of test summaries are found in Supplementary Data 9 and 10.

**Extended Data Fig. 8:**
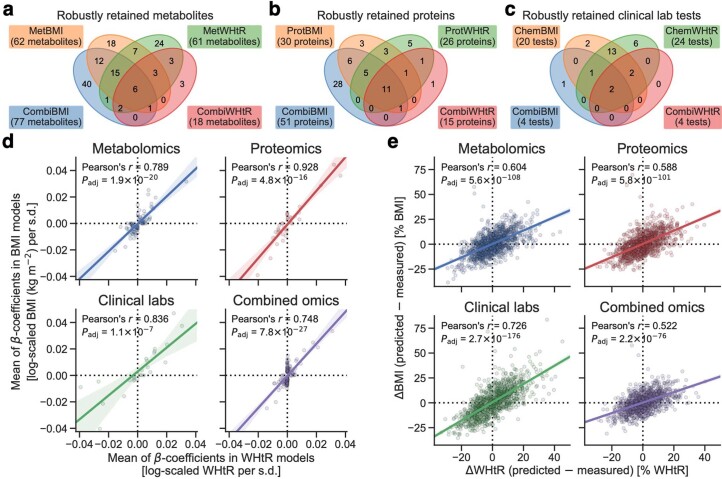
Predominant commonality with minor specificity was observed between the omics-based BMI and WHtR models. **a**–**d**, Comparison of the omics-based LASSO model between BMI and WHtR. **a**–**c**, The number of the variables that were robustly retained across all ten LASSO models. **d**, Correlation of the mean of *β*-coefficients in the ten LASSO models. Only the robustly retained analytes in either BMI models or WHtR models were analyzed. **e**, Correlation between ΔBMI (that is, difference of the omics-inferred BMI from the measured BMI) and ΔWHtR (that is, difference of the omics-inferred WHtR from the measured WHtR). Only the participants having both BMI and WHtR were analyzed. **d**,**e**, The solid line is the OLS linear regression line with 95% confidence interval. *P*_adj_: adjusted *P* value of two-sided Pearson’s correlation test with the Benjamini–Hochberg method across the four categories. *n* = 92 metabolites (**d**, Metabolomics), 36 proteins (**d**, Proteomics), 26 clinical laboratory tests (**d**, Clinical labs), 146 analytes (**d**, Combined omics) or 1,078 participants (**e**). All exact values of test summaries are found in Supplementary Data 10.

Fat distribution in the body is an important feature for understanding the heterogeneous nature of obesity. In particular, abdominal obesity, which is characterized by excessive visceral fat (rather than subcutaneous fat) around the abdominal region, is associated with chronic diseases such as MetS^40^. Thus, we analyzed WHtR, an anthropometric measure of abdominal obesity^41,42^, in the Arivale cohort using the same scheme with the omics-based BMI models (Extended Data Fig. 7a and Methods). The omics-based WHtR models exhibited consistent findings (Extended Data Fig. 7) and characteristics (Extended Data Fig. 8) to the omics-based BMI models. Moreover, in the TwinsUK cohort, DXA measurements of fat in the android region (+_BMI_) were significantly higher in the mismatched group compared to the matched group within the normal BMI class (FDR < 0.05; Extended Data Fig. 6c). Collectively, although classical BMI requires complementary information of the fat distribution for the diagnosis of abdominal obesity, the omics-based BMI model likely captures the obesity characteristics, including abdominal obesity.

### Gut microbiome and omics-inferred BMIs

**Fig. 4:**
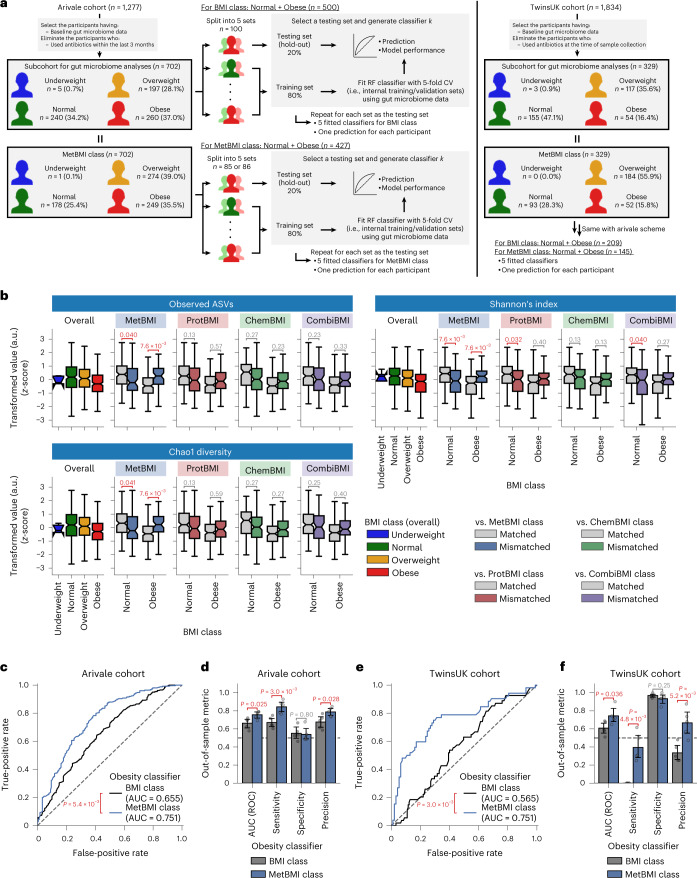
Metabolomics-inferred BMI reflected gut microbiome profiles better than BMI. **a**, Overview of study cohorts and the gut microbiome-based obesity classifier generation. CV, cross-validation; RF, random forest. **b**, Difference in gut microbiome α-diversity between the matched and mismatched groups within the normal or obese BMI class. Each comparison value indicates adjusted *P* value, calculated from OLS linear regression with BMI, sex, age and ancestry principal components as covariates while adjusting multiple testing with the Benjamini–Hochberg method across the 24 (2 BMI classes × 4 omics categories × 3 metrics) regressions. Data: the standard box plot (Methods); *n* = 240 (Normal) or 260 (Obese) participants (see Supplementary Data 6 for each sample size). a.u., arbitrary units. **c**,**e**, ROC curve of the gut microbiome-based model classifying participants to the normal versus obese class in the Arivale (**c**) or TwinsUK (**e**) cohort. Each ROC curve was generated from the overall participants: *n* = 500 (**c**, BMI class), 427 (**c**, MetBMI class), 209 (**e**, BMI class) or 145 (**e**, MetBMI class) participants. The dashed line indicates a random classification line. *P*: *P* value of two-sided unpaired DeLong’s test. **d**,**f**, Comparison of model performance between the BMI and MetBMI classifiers in the Arivale (**d**) or TwinsUK (**f**) cohort. Out-of-sample metric value was calculated from each corresponding hold-out testing set. Data: mean with 95% confidence interval, *n* = 5 models. *P*: *P* value of two-sided Welch’s *t*-test. All exact values of test summaries are found in Supplementary Data 6 and 10.

Given our previous finding that the association between blood metabolites and bacterial diversity is dependent on BMI^20^ and the current finding that the omics-based BMI models capture heterogeneous metabolic health states (Fig. 3), we hypothesized that MetBMI represents gut microbiome α-diversity better than actual BMI. For the 702 Arivale participants who had both stool-derived gut microbiome and blood omic datasets (Fig. 4a and Methods), we examined relationships between gut microbiome α-diversity (the number of observed species, Shannon’s index and Chao1 index) and the omics-based BMI misclassification. The matched and mismatched groups against MetBMI class showed significant differences in all α-diversity metrics within both normal and obese BMI classes (Fig. 4b), with the concordant pattern to the phenotypes that are negatively associated with BMI (Fig. 3d,e), implying that the MetBMI class reflects bacterial diversity better than the standard BMI class. The misclassification against the other omics categories did not show these significant differences for all α-diversity metrics and both BMI classes (Fig. 4b), consistent with our previous observation that plasma metabolomics showed stronger association with gut microbiome structure than either proteomics or clinical labs^20^.

We further examined the predictive power of gut microbiome profiles for MetBMI. For each of the measured BMI and MetBMI classes, we generated models classifying individuals into normal class versus obese class based on gut microbiome 16S rRNA gene amplicon sequencing data, using a five-fold iteration scheme of the random forest algorithm with five-fold cross-validation (Fig. 4a and Methods). Compared to the classifier for the measured BMI class, the classifier for MetBMI class showed significantly larger area under the curve (AUC) in the receiver operator characteristic (ROC) curve in the Arivale cohort (AUC = 0.66 (BMI) and 0.75 (MetBMI); Fig. 4c), with significantly higher sensitivity and precision (Fig. 4d). Moreover, by applying the same scheme to the stool-derived whole metagenomic shotgun sequencing (WMGS) data of the 329 TwinsUK participants^43^ (Fig. 4a and Methods), we validated the significant outperformance of the MetBMI classifier in the TwinsUK cohort (AUC = 0.57 (BMI) and 0.75 (MetBMI); Fig. 4e,f). These classifiers were generated again for the TwinsUK cohort (instead of using the classifiers that were fitted to the Arivale dataset; Fig. 4a) owing to the difference in sequencing methods (amplicon sequencing versus WMGS) while considering that the TwinsUK participants’ MetBMIs were predicted from the Arivale-fitted MetBMI models (Fig. 1a). These findings suggest that, although other factors (such as dietary intake^19^) may be involved, MetBMI has a stronger correspondence to gut microbiome features than classical BMI.

### Responses of omics-inferred BMIs to a lifestyle intervention

**Fig. 5:**
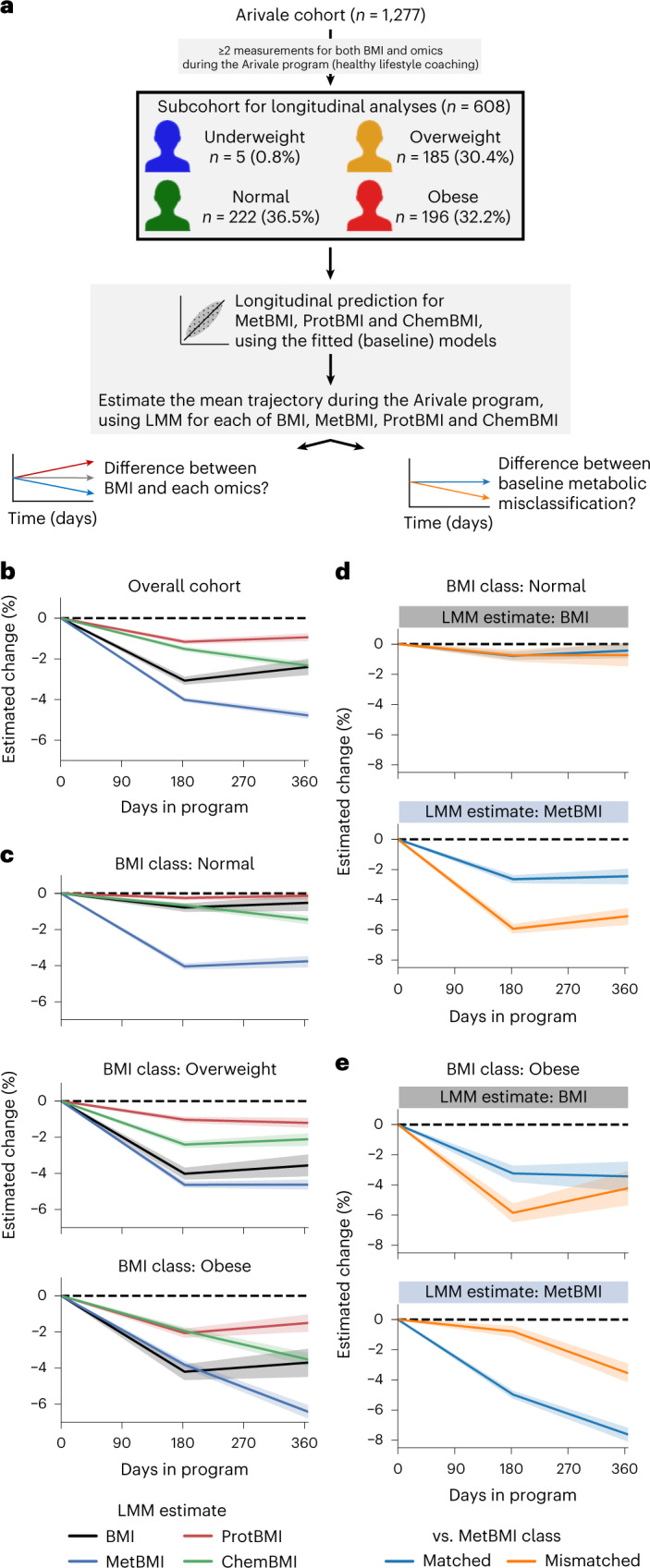
Metabolic health of the metabolically obese group was improved during a healthy lifestyle intervention program. **a**, Overview of the longitudinal analysis using omics-inferred BMI. **b**,**c**, Longitudinal change in the omics-inferred BMI within the overall cohort (**b**) or within each baseline BMI class (**c**). Average trajectory of each measured or omics-inferred BMI was independently estimated using LMM with random effects for each participant (Methods) in the overall cohort (**b**) or in each baseline BMI class-stratified group (**c**). **d**,**e**, Longitudinal change in MetBMI of the misclassified participants within the normal (**d**) or obese (**e**) BMI class. Average trajectory of each BMI or MetBMI was independently estimated using the above LMM with the baseline misclassification of BMI class against MetBMI class as additional fixed effects (Methods) in each baseline BMI class-stratified group. **b**–**e**, The dashed line corresponds to the baseline value of each estimate. Data: mean with 95% confidence interval; *n* = 608 (**b**), 222 (**c**, Normal), 185 (**c**, Overweight), 196 (**c**, Obese), 137 (**d**, Matched), 85 (**d**, Mismatched), 139 (**e**, Matched) or 57 (**e**, Mismatched) participants.

Longitudinal changes in omic profiles during the Arivale program were investigated in a subcohort of 608 participants based on the available longitudinal measurements (Fig. 5a and Methods). Given the participant-dependent variability in both count and timepoint of data collections, we estimated the average trajectory of each measured or omics-inferred BMI in the Arivale subcohort using a linear mixed model (LMM) with random effects for each participant (Methods). Consistent with previous analysis^25,44^, the mean BMI estimate for the overall cohort decreased during the program (Fig. 5b). The decrease of MetBMI was larger than that of measured BMI, whereas the decrease of ProtBMI was minimal and even smaller than that of measured BMI (Fig. 5b), suggesting that plasma metabolomics is highly responsive to the lifestyle intervention in the short term, whereas proteomics (measured from the same blood draw) is more resistant to change during the same intervention period. Subsequently, we generated LMMs with the baseline BMI class stratification. The mean estimates of the measured BMI, ProtBMI and ChemBMI exhibited negative changes over time in the overweight and obese BMI classes but not in the normal BMI class (Fig. 5c). In contrast, the mean MetBMI estimate exhibited a significant decrease across all BMI classes (Fig. 5c), suggesting that metabolomics data capture information about the metabolic health response to the lifestyle intervention, beyond the baseline BMI class and the changes in actual BMI and other omic profiles.

Given the existence of multiple metabolic health substates within the standard BMI classes (Fig. 3), we further investigated the difference between misclassification strata against the baseline MetBMI class. In the (baseline) normal BMI class, whereas the mean estimate of the measured BMI remained constant in both matched and mismatched groups, the mean MetBMI estimate exhibited larger reduction in the mismatched group than the matched group (Fig. 5d), suggesting that the participants with MUNW phenotype improved their metabolic health to a greater extent than the participants with MHNW phenotype. Likewise, in the (baseline) obese BMI class, whereas the decrease in the mean estimate of the measured BMI was not different between the matched and mismatched groups (at 1 year after the enrollment), the decrease in the mean MetBMI estimate was larger in the matched group than in the mismatched group (Fig. 5e), suggesting that the participants with MUO phenotype improved their metabolic health to a greater extent than the participants with MHO phenotype. These results suggest that metabolic health was substantially improved during the program, in accordance with an individual’s baseline metabolomic state rather than with the individual’s baseline BMI class.

### Blood analyte network dynamics and MetBMI class

**Fig. 6:**
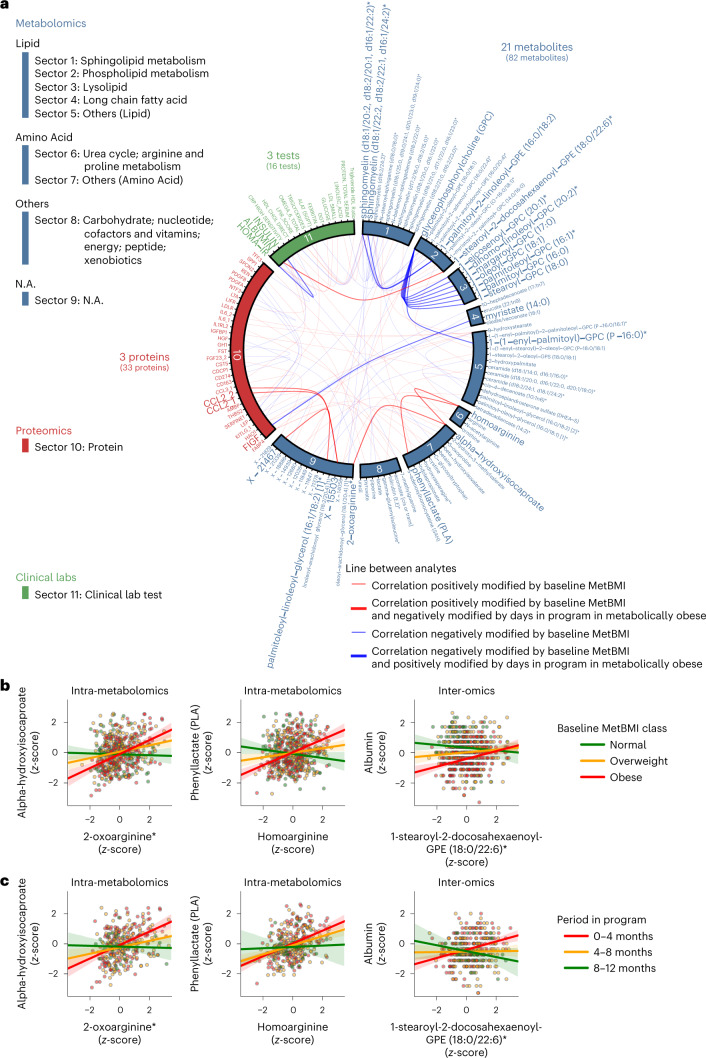
Plasma analyte correlation network in the metabolically obese group shifted toward a structure observed in a metabolically healthier state during a healthy lifestyle intervention program. **a**, Cross-omic interactions modified by MetBMI and days in the program. For each of the 608,856 pairwise relationships of plasma analytes (766 metabolites, 274 proteomics and 64 clinical laboratory tests), the baseline relationship between analyte–analyte pair and MetBMI within the Arivale subcohort (Fig. 5a; 608 participants) was assessed using their interaction term in each GLM (Methods) while adjusting multiple testing with the Benjamini–Hochberg method. The 100 analyte–analyte pairs (82 metabolites, 33 proteins and 16 clinical laboratory tests) that were significantly modified by the baseline MetBMI are presented. For each of these 100 pairs, the longitudinal relationship between analyte–analyte pair and days in the program within the metabolically obese group (that is, the baseline obese MetBMI class; 182 participants) was further assessed using their interaction term in each GEE (Methods) while adjusting multiple testing with the Benjamini–Hochberg method. The 27 analyte–analyte pairs (21 metabolites, three proteins and three clinical laboratory tests) that were significantly modified by days in the program are highlighted by line width and label font size. N.A., not available. All exact values of test summaries are found in Supplementary Data 7. **b**,**c**, Representative examples of the analyte–analyte pair that was significantly modified by both baseline MetBMI (**b**) and days in the program (**c**) in **a**. The solid line in each panel is the OLS linear regression line with 95% confidence interval. *n* = 530 (**b**, left), 553 (**b**, center) or 566 (**b**, right) participants; *n* = 324 (**c**, left), 339 (**c**, center) or 347 (**c**, right) measurements from the 182 participants of the metabolically obese group. Of note, data points outside of plot range are trimmed in these presentations.

We explored longitudinal changes in plasma analyte correlation networks, focusing on the metabolically obese group defined by MetBMI class. Based on the importance of the baseline metabolomic state (Fig. 5d,e), we first assessed relationships between each plasma analyte–analyte correlation and the baseline MetBMI within the Arivale subcohort (Fig. 5a; 608 participants), using their interaction term in a generalized linear model (GLM) of each analyte–analyte pair (Methods). In this type of model, the statistical test assesses whether the relationship between any two analytes is dependent on a third variable (in this case, the baseline MetBMI). Among 608,856 pairwise relationships of plasma analytes, 100 analyte–analyte correlation pairs, comprising 82 metabolites, 33 proteins and 16 clinical laboratory tests, were significantly modified by the baseline MetBMI (FDR < 0.05; Supplementary Data 7). Subsequently, we assessed longitudinal changes of these 100 pairs within the baseline obese MetBMI class (182 participants), using the interaction term (that is, interaction with days in the program) in a generalized estimating equation (GEE) of each analyte–analyte pair (Methods). Among the 100 pairs, 27 analyte–analyte correlation pairs were significantly modified by days in the program (FDR < 0.05; Fig. 6a). These 27 pairs were mainly derived from metabolites (21 metabolites, three proteins and three clinical laboratory tests). One of these time-varying pairs was homoarginine and phenyllactate (PLA). Homoarginine was found to be a biomarker for CVD^45^ and was a robustly retained positive predictor in MetBMI and CombiBMI models (Fig. 2a and Extended Data Fig. 5a). PLA is a gut microbiome-derived phenylalanine derivative known to have antimicrobial activity and antioxidant activity^46,47^. The positive association between homoarginine and PLA was observed in the obese MetBMI class at baseline (Fig. 6b) and became weaker in this class during the course of the intervention (Fig. 6c), implying that metabolic dysregulation specific to the metabolically obese group was somewhat improved during the program. These findings indicate that metabolic improvement was not limited to changes in specific blood analyte concentrations but also changes in the association structure among analytes.

## Discussion

Obesity is a significant risk factor for many chronic diseases^3–6^. The heterogeneous nature of human health conditions, with variable manifestations ranging from metabolic abnormalities to cardiovascular symptoms, calls for deeper molecular characterizations to optimize wellness and reduce the current global epidemic of chronic diseases. In this study, we demonstrated that obesity perturbs human physiology, as reflected across all the studied omics modalities. Machine learning-based multiomic BMI estimates were better suited to identifying heterogeneous metabolic health and gut microbiome structure than actual BMI while maintaining a high level of interpretability and intuitiveness attributed to the original metric. Plasma metabolomics exhibited the strongest (and/or earliest) response to lifestyle coaching, whereas plasma proteomics exhibited a weaker (and/or more delayed) response than actual BMI. Compared to the participants with metabolically healthy phenotype (that is, BMI class ≥ MetBMI class), the participants with metabolically unhealthy phenotype (that is, BMI class < MetBMI class) exhibited a greater improvement in their metabolic health (but not in weight loss per se) in response to the healthy lifestyle coaching. Dozens of analyte–analyte associations were modified in the participants of the metabolically obese group (that is, obese MetBMI class), after the healthy lifestyle intervention.

Although many observational studies have explored proteins and metabolites as biomarkers for obesity^5,6,23,48–50^, each biomarker usually reflects a specific aspect (or population average) of obesity, and relationships between the biomarkers remain to be elucidated. In contrast, the omics-based BMI models automatically incorporated well-known biomarkers and, hence, can be regarded as multidimensional profiles of obesity. Furthermore, we observed analytes that were associated with a small proportion of the variance in BMI while being strong predictive features in the omics-based BMI models—for example, RAGE, which has been highlighted in the contexts of T2DM and CVD^51^. Therefore, the omics-based BMI models may reflect not only the mechanistic information of obesity but also the early transition toward clinical manifestations of obesity-related chronic diseases.

A previous study investigating multiomic changes in response to weight perturbations demonstrated that some weight gain-associated blood signatures were reversed during subsequent weight loss while others persisted^52^. We found that MetBMI was more responsive to the healthy lifestyle intervention than actual BMI or ChemBMI, whereas ProtBMI was more resistant to the same intervention. Our analyses on the predictors of the omics-based BMI models suggested that the distribution of feature importance among metabolites was wider, whereas only a small subset of measured proteins (~5 proteins) was predominantly reflective of obesity profiles. Therefore, the effect of lifestyle coaching may consist of small additive contributions in blood metabolites in the short term. However, longer longitudinal analyses are needed to infer the physiological meaning of these omics-dependent dynamics. It is possible that ProtBMI shows a delayed response to the intervention, indicating that blood metabolites and proteins may be early and late responders to a lifestyle intervention, respectively, such as the relationship between blood glucose and HbA1c in the assessment of glucose homeostasis^53^. If the difference between the measured and omics-inferred BMIs remains constant even after 1 year, blood metabolites and proteins could be more and less sensitive to a lifestyle intervention than classical BMI, respectively. As a translational implication, monitoring blood multiomics during weight loss programs would help participants maintain their motivation to stay engaged with persistent lifestyle changes, because they would receive rapid feedback on how lifestyle changes were impacting their health, even in the absence of weight loss.

Our study had several limitations. The analytes that were retained in the omics-based models do not necessarily have causal relationships with obesity phenotypes. These relationships could be indicative of obesity or affected by other factors that were not included in the models. Our measurements did not cover all biomolecules in blood; in particular, proteomics was based on three targeted Olink panels. Thus, our findings on metabolomic and proteomic states are restricted to the analytes that we could measure. This study was not designed as a randomized controlled trial, and we cannot strictly evaluate the effectiveness of the lifestyle intervention (for example, bigger improvements in the obese group compared to the normal-weight group may be due to the regression-toward-the-mean effect^44^). In addition, we used time as the variable in longitudinal analyses under an assumption that the program enrollment itself affected participants’ BMI and omic profiles. If we had more detailed data on the intervention (for example, magnitude and participant compliance), we would be able to improve the assessment of its effect. The generalizability of our findings may be limited, because this study was an observational study of largely White individuals from the Pacific West of the United States and from the United Kingdom, and validation with an external cohort relied on the female-dominated cohort (96.7%) and its metabolomics data.

In summary, this study highlights the usefulness of blood multiomic profiling for predictive and preventive medicine. It also outlines an unprecedented multiomic characterization of obesity and will serve as a valuable resource for characterizing metabolic health and identifying actionable targets for health management.

## Methods

### Arivale cohort

The main study cohort was derived from 6,223 individuals who participated in a wellness program offered by a currently closed commercial company (Arivale, Inc.) between 2015 and 2019. An individual was eligible for enrollment if the individual was over 18 years of age, not pregnant and a resident of any US state except New York; participants were primarily recruited from Washington, California and Oregon. The participants were not screened for any particular disease. During the Arivale program, each participant was provided personalized lifestyle coaching via telephone by registered dietitians, certified nutritionists or registered nurses. This coaching was designed to improve the participant’s health based on the combination of clinical laboratory tests, genetic predispositions and published scientific evidence; for example, reduction of sodium intake might be recommended to any participants with high blood pressure, but if they also had risk alleles indicating enhanced susceptibility to dietary sodium, this risk would be emphasized (see a previous report^25^ for more details).

In the current study, to compare the associations between BMI and host phenotypes across different omics, we limited the original cohort to the participants whose datasets contained (1) all main omic measurements (metabolomics, proteomics and clinical laboratory tests) from the same first blood draw; (2) a BMI measurement within ±1.5 months from the first blood draw; and (3) genetic information (for using as covariates). We also eliminated (1) outlier participants whose baseline BMI was beyond ±3 s.d. from the mean in the baseline BMI distribution and (2) participants whose any of omic datasets contained more than 10% missingness in the filtered analytes (see the ‘data cleaning’ subsection). The final Arivale cohort consisted of 1,277 (821 female and 456 male) participants (Fig. 1a) who exhibited consistent demographics (Extended Data Fig. 1a–c and Supplementary Data 1) with the study cohorts defined in the previous Arivale studies^20,25–29^. For the analyses of gut microbiome, subcohort was defined with the 702 (486 female and 216 male) participants from the Arivale cohort who collected a stool sample within ±1.5 months from the first blood draw and did not use antibiotics in the last 3 months (Fig. 4a and Supplementary Data 1). For longitudinal analyses, subcohort was defined with the 608 (410 female and 198 male) participants from the Arivale cohort whose datasets contained two or more time-series datasets for both BMI and omics during 18 months after enrollment (Fig. 5a and Supplementary Data 1). For the analyses of WHtR, subcohort was defined with the 1,078 (689 female and 389 male) participants from the Arivale cohort whose datasets contained the baseline WHtR measurement within ±1.5 months from the first blood draw and within ±3 s.d. from the mean in the baseline WHtR distribution (Extended Data Fig. 7a and Supplementary Data 1).

### TwinsUK cohort

The external cohort was derived from 17,630 individuals who participated in the TwinsUK Registry, a British national register of adult twins^31^. Twins were recruited as volunteers by media campaigns without screening for any particular disease. The participants had two or more clinical visits for biological sampling between 1992 and 2022. In the current study, to validate our findings in the Arivale cohort, we limited the original cohort to the participants whose datasets contained all measurements for metabolomics^32^, BMI and the obesity-related standard clinical measures (that is, defined by triglycerides, HDL cholesterol, LDL cholesterol, glucose, insulin and HOMA-IR throughout the current study) from the same visit. We also eliminated (1) outlier participants whose BMI was beyond ±3 s.d. from the mean in the overall BMI distribution and (2) participants whose metabolomic dataset contained more than 10% missingness in the filtered metabolites (see the ‘data cleaning’ subsection). The final TwinsUK cohort consisted of 1,834 (1,774 female and 60 male) participants (Fig. 1a, Extended Data Fig. 1d–f and Supplementary Data 1). For the analyses of gut microbiome, subcohort was defined with the 329 (307 female and 22 male) participants from the TwinsUK cohort who collected a stool sample within ±1.5 months from the clinical visit and did not use antibiotics at that time (Fig. 4a and Supplementary Data 1).

### Ethics statement

The current study was conducted with de-identified data of the participants who had consented to the use of their anonymized data in research. Procedures were run under the Western Institutional Review Board (study numbers 20170658 at the Institute for Systems Biology and 1178906 at Arivale). Application of data access for the TwinsUK cohort was approved by the TwinsUK Resource Executive Committee (project number E1192).

### Data collections and data cleaning for the Arivale cohort

Multiomics data for the Arivale participants included genomics and longitudinal measurements of metabolomics, proteomics, clinical laboratory tests, gut microbiomes, wearable devices and health/lifestyle questionnaires. Peripheral venous blood draws for all measurements were performed by trained phlebotomists at LabCorp (Laboratory Corporation of America Holdings) or Quest (Quest Diagnostics) service centers. Saliva to measure analytes such as diurnal cortisol and dehydroepiandrosterone was sampled by participants at home using a standardized kit (ZRT Laboratory). Stool samples for gut microbiome measurements were obtained by participants at home using a standardized kit (DNA Genotek).

- Genomics
  DNA was extracted from each whole blood sample and underwent whole-genome sequencing (1,257 participants) or single-nucleotide polymorphism (SNP) microarray genotyping (20 participants). Genetic ancestry was calculated with principal components using a set of ~100,000 ancestry-informative SNP markers, as described previously^25^. PRSs were constructed using publicly available summary statistics from published genome-wide association studies, as described previously^27^.
- Blood-measured omics
  Metabolomics data were generated by Metabolon using ultra-high-performance liquid chromatography–tandem mass spectrometry (UHPLC–MS/MS) for plasma derived from each whole blood sample. Proteomics data were generated using proximity extension assay for plasma derived from each whole blood sample with several Olink target panels (Olink Proteomics), and only the measurements with the Cardiovascular II, Cardiovascular III and Inflammation panels were used in the current study because the other panels were not necessarily applied to all samples. All clinical laboratory tests were performed by LabCorp or Quest in a Clinical Laboratory Improvement Amendments-certified lab, and only the measurements by LabCorp were selected in the current study to eliminate potential differences between vendors. In the current study, the batch-corrected datasets with in-house pipeline were used, and the metabolomic dataset was log_*e*_-transformed. In addition, analytes missing in more than 10% of the baseline samples were removed from each omic dataset, and observations missing in more than 10% of the remaining analytes were further removed. The final filtered metabolomics, proteomics and clinical labs consisted of 766 metabolites, 274 proteins and 71 clinical laboratory tests, respectively (Supplementary Data 2).
- Gut microbiome
  Gut microbiome data were generated based on 16S amplicon sequencing of the V3+V4 region using a MiSeq sequencer (Illumina) for DNA extracted from each stool sample, as previously described^28^. In brief, the FASTQ files were processed using the mbtools workflow (version 0.37.1; https://github.com/Gibbons-Lab/mbtools) to remove noise, infer amplicon sequence variants (ASVs) and remove chimeras. Taxonomy assignment was performed using the SILVA ribosomal RNA gene database (version 132)^54^. In the current study, the final collapsed ASV table across the samples consisted of 394, 341, 85, 45, 26 and 16 taxa for species, genus, family, order, class and phylum, respectively. Gut microbiome α-diversity was calculated at the ASV level using Shannon’s index calculated by ${H = - {\mathop {\sum}\nolimits_{i = 1}^S {p_i}}{\rm {ln}} {p_i}}$, where *p*_*i*_ is the proportion of a community *i* represented by ASVs, or using Chao1 diversity score calculated by $S_{{{{\mathrm{Chao1}}}}} = S_{{{{\mathrm{obs}}}}} + {\textstyle{n_1^2} \over {2n_2}}$, where *S*_obs_ is the number of observed ASVs; *n*_1_ is the number of singletons (ASVs captured once); and *n*_2_ is the number of doubletons (ASVs captured twice).
- Anthropometrics, saliva-measured analytes and daily physical activity measures
  Anthropometrics, including weight, height, waist circumference and blood pressure, were measured at the time of blood draw and also reported by participants, which generated diverse timing and numbers of observations depending on each participant. BMI and WHtR were calculated from the measured anthropometrics with the weight divided by squared height (kg m^−2^) and the waist circumference divided by height (unit-less), respectively. Measurements of saliva samples were performed in the testing laboratory of ZRT Laboratory. Daily physical activity measures, such as heart rate, moving distance, step count, burned calories, floors climbed and sleep quality, were tracked using the Fitbit wearable device. To manage variations between days, monthly averaged data were used for these daily measures. In the current study, the baseline measurement for these longitudinal measures was defined with the closest observation to the first blood draw per participant and data type, and each dataset was eliminated from analyses when its baseline measurement was beyond ±1.5 months from the first blood draw.

### Data collections and data cleaning for the TwinsUK cohort

Data resource for the TwinsUK participants included longitudinal measurements of metabolomics, clinical laboratory tests, DXA and health/lifestyle questionnaires^31^. The necessary datasets for the current study were provided by the Department of Twin Research & Genetic Epidemiology (King’s College London). In the current study, after each provided dataset was cleaned as follows, the earliest visit among the visits from which all of metabolomics, BMI and standard clinical measures had been measured was defined as the baseline visit for each participant. As an exception, the later visit among them was prioritized as the baseline visit if the participant had gut microbiome data within ±1.5 months from the visit. Only the baseline visit measurements were analyzed.

- Blood-measured metabolomics
  Metabolomics data were originally generated by Metabolon using UHPLC–MS/MS for each serum sample^32^. In the current study, the provided median-normalized dataset was log_*e*_-transformed. In addition, metabolites missing in more than 10% of the overall samples were removed from the metabolomic dataset, and observations missing in more than 10% of the remaining metabolites were further removed. The final filtered metabolomics consisted of 683 metabolites.
- BMI
  In the current study, the BMI values that had been already calculated and included in the provided metabolomics data file were used.
- Standard clinical measures and other phenotypic measures
  In the current study, because the provided phenotypic datasets contained multiple measurements for a phenotype even from a single visit of a participant (for example, owing to project difference or repeated measurements), multiple measurements were flattened into a single measurement for a phenotype per each participant’s visit by taking the mean value. During this flattening step, the difference in unit was properly adjusted, and the value indicating below detection limit was regarded as 0. HOMA-IR was calculated from the datasets of glucose, insulin and fasting condition with the formula: HOMA-IR = fasting glucose (mmol L^−1^) × fasting insulin (mIU L^−1^) × 22.5^−1^.
- Gut microbiome
  Gut microbiome data were originally generated based on WMGS using a HiSeq 2500 sequencer (Illumina) for DNA extracted from each stool sample^43^. In the current study, the raw sequencing data were obtained from the National Center for Biotechnology Information (NCBI) Sequence Read Archive (PRJEB32731) and applied to a processing pipeline on Nextflow (version 22.04.5; https://github.com/Gibbons-Lab/pipelines). Through this pipeline, the obtained FASTQ files were processed using the fastp (version 0.23.2) tool^55^ to filter and trim the reads, and taxonomic abundance was obtained using the Kraken 2 (version 2.1.2) and Bracken (version 2.6.0) tools^56^ with the Kraken 2 default database (based on NCBI RefSeq). The final collapsed taxonomic table across the samples consisted of 4,669, 1,225, 354, 167, 76 and 35 taxa for species, genus, family, order, class and phylum, respectively.

### Blood omics-based BMI and WHtR models

For each Arivale baseline omic dataset, missing values were first imputed with a random forest algorithm using the Python missingpy (version 0.2.0) library (corresponding to R MissForrest package^57^). For sex-stratified models (Extended Data Fig. 2d), the datasets after imputation were divided into sex-stratified datasets. Subsequently, the values in each omic dataset were standardized with *z*-score using the mean and s.d. per analyte. Then, ten iterations of LASSO modeling with ten-fold cross-validation (Fig. 1a and Extended Data Fig. 7a) were performed for the (unstandardized) log_*e*_-transformed BMI or WHtR and each processed omic dataset, using the LassoCV application programming interface (API) of the Python scikit-learn (version 1.0.1) library. Training and testing (hold-out) sets were generated by splitting participants into ten sets with one set as a testing (hold-out) set and the remaining nine sets as a training set and iterating all combinations over those ten sets; that is, overfitting was controlled using ten-fold iteration with ten testing (hold-out) sets, and hyperparameter was decided using ten-fold cross-validation with internal training and validation sets from each training set. Consequently, this procedure generated ten fitted sparse models for each omics category (Supplementary Data 3 and 8) and one single testing (hold-out) set-derived prediction from each omics category for each participant. The same modeling scheme while replacing LASSO with elastic net, ridge or random forest was performed using Python scikit-learn ElasticNetCV, RidgeCV or RandomForestRegressor-implemented GridSearchCV API, respectively. In this random forest modeling, the number of trees in the forest and the number of features were set as the hyperparameters to be decided through cross-validation. For the standard measures-based models, the above modeling scheme was applied to OLS linear regression with sex, age, triglycerides, HDL cholesterol, LDL cholesterol, glucose, insulin and HOMA-IR as regressors, using Python scikit-learn LinearRegression API. Of note, ten split sets were fixed among the omics categories and the modeling methods, and no significant difference in BMI, WHtR, sex, age and ancestry principal components 1–5 among those ten sets was confirmed, using Pearson’s *χ*^2^ test for categorical variables and ANOVA for numeric variables while adjusting multiple testing with the Benjamini–Hochberg method across the tested variables (Supplementary Data 1).

For the TwinsUK cohort, the metabolomic dataset was applied to the random forest imputation, and then each dataset of metabolomics and standard clinical measures was applied to *z*-score standardization as well as the Arivale datasets. Using the ten LASSO or OLS linear regression models that were fitted by the Arivale dataset, one single prediction was calculated from each processed dataset for each participant by taking the mean of ten predicted values. For metabolomics, the ten MetBMI models were generated again but restricting the input Arivale metabolomics to the common 489 metabolites in the Arivale and TwinsUK panels (Extended Data Fig. 3).

For the LASSO-modeling iteration analysis (Extended Data Figs. 2e–h and 7f–i), ten LASSO models were repeatedly generated with the above modeling scheme. At the end of each iteration, the variable that was retained across ten models and that had the highest absolute value for the mean of ten *β*-coefficients was removed from the input omic dataset.

For longitudinal predictions of the Arivale subcohort, one single prediction at a timepoint was calculated from each processed time-series omic dataset for each participant, using the baseline LASSO model for which the participant was included in the baseline testing (hold-out) set. This was because (1) the baseline measurements were minimally affected by the personalized lifestyle coaching; (2) both count and timepoint of data collections were different among the participants; and (3) potential data leakage might be derived from the relationships between the baseline and following measurements for the same participant. For processing, each time-series omic dataset was applied to two-step random forest imputation; that is, the baseline missingness was first imputed based on the baseline data structure, and the remaining missingness was next imputed based on the overall data structure. Each imputed dataset was subsequently applied to *z*-score standardization using the mean and s.d. in the baseline distribution.

Model performance was conservatively evaluated by the out-of-sample *R*^2^ that was calculated from each corresponding hold-out testing set in the Arivale cohort or from the external testing set in the TwinsUK cohort. Pearson’s *r* between the measured and predicted values was calculated from the overall participants of the Arivale or TwinsUK cohort. Difference of the predicted value from the measured value (ΔMeasure; that is, ΔBMI or ΔWHtR) was calculated with (the predicted value − the measured value) × (the measured value)^−1^ × 100 (that is, the unit of ΔMeasure was (% Measure)). In the random forest model, the importance of a feature was calculated as the normalized total reduction of the mean squared error that was brought by the feature.

### Health classification

Each participant was classified using each of the measured and omics-inferred BMIs based on the WHO international standards for BMI cutoffs (underweight: <18.5 kg m^−2^, normal: 18.5–25 kg m^−2^, overweight: 25–30 kg m^−2^, obese: ≥30 kg m^−2^)^12^. For the misclassification of BMI class against the omics-inferred BMI class, each participant was categorized into either a matched or a mismatched group when the measured BMI class was matched or mismatched to each omics-inferred BMI class, respectively.

For a clinically defined metabolic health classification, the participants having two or more MetS risks of the National Cholesterol Education Program Adult Treatment Panel III guidelines were judged as the metabolically unhealthy group, whereas the other participants were judged as the metabolically healthy group^34,35^. Concretely, the MetS risk components were (1) systolic blood pressure ≥130 mm Hg, diastolic blood pressure ≥85 mm Hg or using anti-hypertensive medication; (2) fasting triglyceride level ≥150 mg dl^−1^; (3) fasting HDL cholesterol level <50 mg dl^−1^ for female and <40 mg dl^−1^ for male or using lipid-lowering medication; and (4) fasting glucose level ≥100 mg dl^−1^ or using anti-diabetic medication. Only the participants who had all these information were assessed in the corresponding analyses (Fig. 3b and Extended Data Figs. 6a and 7m).

### Gut microbiome-based models for classifying obesity

For the Arivale gut microbiome dataset, the whole ASV table (907 taxa from species to phylum) was pre-processed (that is, positively shifted by 1, log_*e*_-transformed and standardized with *z*-score using the mean and s.d. per taxon) and then applied to dimensionality reduction using PCA API of the Python scikit-learn (version 1.0.1) library; the projected values onto the first 50 principal components (0.4–5.1% variance explained) were supplied as the input gut microbiome features. Two types of classifiers were trained on these gut microbiome features: one predicting whether an individual is obese BMI class and the other predicting whether an individual is obese MetBMI class. Both models were independently constructed through a five-fold iteration scheme of random forest with five-fold cross-validation (Fig. 4a) using Python scikit-learn RandomForestClassifier-implemented GridSearchCV API. In this random forest modeling, the number of trees in the forest and the number of features were set as the hyperparameters to be decided through cross-validation. Training and testing (hold-out) sets were generated by splitting the participants of the normal and obese classes into five sets, with one set as a testing (hold-out) set and the remaining four sets as a training set, and iterating all combinations over those five sets; that is, overfitting was controlled using five-fold iteration with five testing (hold-out) sets, and hyperparameters were decided using five-fold cross-validation with internal training and validation sets from each training set. Consequently, this procedure generated five fitted classifiers for each BMI or MetBMI class and one single testing (hold-out) set-derived prediction from each classifier type for each participant. Note that this prediction included two types: either normal or obese class by a vote of the trees (that is, binary prediction) and the mean probability of obese class among the trees.

For the TwinsUK gut microbiome dataset, the whole taxonomic table (6,526 taxa from species to phylum) was pre-processed and then applied to dimensionality reduction as well as the Arivale dataset; the projected values onto the first 50 principal components (0.2–40.1% variance explained) were supplied as the input gut microbiome features. Then, the five obesity classifiers for each BMI or MetBMI class were generated as well as the above Arivale procedure, and one single testing (hold-out) set-derived prediction from each classifier type was calculated for each participant (Fig. 4a).

Model performance of each classifier was conservatively evaluated using each corresponding hold-out testing set. AUC in the ROC curve and the average precision were calculated using the probability predictions, whereas sensitivity and specificity were calculated from the confusion matrix using the binary predictions. The overall ROC curve and its AUC were calculated from all the participants’ probability predictions, using the R pROC (version 1.18.0) package^58^.

### Longitudinal changes in the measured and omics-inferred BMIs

An LMM was generated for each log_*e*_-transformed measured or omics-inferred BMI in the Arivale subcohort, following the previous approach^25^. As fixed effects regarding time, linear regression splines with knots at 0, 6, 12 and 18 months were applied to days in the program to fit time as a continuous variable rather than a categorical variable, because both count and timepoint of data collections were different among the participants. In addition to the linear regression splines of time as fixed effects, the LMM included sex, baseline age, ancestry principal components 1–5 and meteorological seasons as fixed effects (to adjust potential confounding effects) and random intercepts and random slopes of days in the program as random effects for each participant. Additionally, the same LMM for each measured or omics-inferred BMI was independently generated from each baseline BMI class-stratified group. Of note, this stratified LMM was not generated from the underweight group because its sample size was too small for convergence. For comparing difference among the misclassification strata against the baseline MetBMI class, the above LMM while adding additional fixed effects (the categorical baseline misclassification of BMI class against MetBMI class (that is, binary for the matched versus mismatched) and its interaction terms with the linear regression splines of time) was generated for each measured BMI or MetBMI from each baseline BMI class-stratified group. All LMMs were modeled using MixedLM API of the Python statsmodels (version 0.13.0) library.

### Plasma analyte correlation network analysis

Before the analysis, outlier values that were beyond ±3 s.d. from the mean in the Arivale subcohort baseline distribution were eliminated from the dataset per analyte, and seven clinical laboratory tests, which became almost invariant across the participants, were eliminated from analyses, allowing convergence in the following modeling. Per each analyte, values were converted with a transformation pipeline producing the lowest skewness (for example, no transformation, the logarithm transformation for right-skewed distribution or the square root transformation with mirroring for left-skewed distribution) and standardized with *z*-score using the mean and s.d.

Against 608,856 pairwise combinations of the analytes (766 metabolites, 274 proteomics and 64 clinical laboratory tests), GLMs for the baseline measurements of the Arivale subcohort (Fig. 5a; 608 participants) were independently generated with the Gaussian distribution and identity link function using glm API of the Python statsmodels (version 0.13.0) library. Each GLM consisted of an analyte as a dependent variable, another analyte and the baseline MetBMI as independent variables (with their interaction term) and sex, baseline age and ancestry principal components 1–5 as covariates. The analyte–analyte correlation pair that was significantly modified by the baseline MetBMI was obtained based on the *β*-coefficient (two-sided *t*-test) of the interaction term between the independent variables in GLM while adjusting multiple testing with the Benjamini–Hochberg method (FDR < 0.05).

Against the significant 100 pairs from the GLM analysis (82 metabolites, 33 proteins and 16 clinical laboratory tests; Supplementary Data 7), GEEs for the longitudinal measurements of the metabolically obese group (that is, the baseline obese MetBMI class; 182 participants) were independently generated with the exchangeable covariance structure using Python statsmodels GEE API. Each GEE consisted of an analyte as a dependent variable, another analyte and days in the program as independent variables (with their interaction term) and sex, baseline age, ancestry principal components 1–5 and meteorological seasons as covariates. The analyte–analyte correlation pair that was significantly modified by days in the program was obtained based on the *β*-coefficient (two-sided *t*-test) of the interaction term between the independent variables in GEE while adjusting multiple testing with the Benjamini–Hochberg method (FDR < 0.05).

### Statistical analysis

All data pre-processing and statistical analyses were performed using Python NumPy (version 1.18.1 or 1.21.3), pandas (version 1.0.3 or 1.3.4), SciPy (version 1.4.1 or 1.7.1) and statsmodels (version 0.11.1 or 0.13.0) libraries, except for using the R pROC (version 1.18.0) package^58^ for DeLong’s test^59^. All statistical tests were performed using a two-sided hypothesis. In all cases of multiple testing, *P* values were adjusted with the Benjamini–Hochberg method. Of note, because some hypotheses were not completely independent (for example, hypotheses between combined omics and each individual omics and hypotheses among glucose, insulin and HOMA-IR), this simple *P* value adjustment was regarded as a conservative approach. Significance was based on *P* < 0.05 for single testing and FDR < 0.05 for multiple testing. Test summaries (for example, sample size, degree of freedom, test statistic and exact *P* value) are found in Supplementary Data 4–6, 9 and 10.

Correlations (Figs. 1b and 3a and Extended Data Figs. 3b–d, 4b,f, 7c,d,l and 8d,e) were independently assessed using Pearson’s correlation test (Python SciPy pearsonr API) (with the *P* value adjustment if multiple testing). Comparisons of model performance (Figs. 1c,d and 4d,f and Extended Data Figs. 2d, 4a and 7e) were independently assessed using Welch’s *t*-test (Python statsmodels ttest_ind API) (with the *P* value adjustment if multiple testing). Comparison of overall ROC curves (Fig. 4c,e) was assessed using unpaired DeLong’s test^59^.

In all regression analyses, only the baseline datasets were used, and, unless otherwise specified, all numeric variables were centered and scaled in advance. For the Arivale datasets of anthropometrics, saliva-measured analytes, daily physical activity measures and PRSs, (1) outlier values that were beyond ±3 s.d. from the mean in the cohort distribution were eliminated from the dataset per variable; (2) variables that became almost invariant across the participants were eliminated from the datasets; (3) values were converted with a transformation pipeline producing the lowest skewness (for example, no transformation, the logarithm transformation for right-skewed distribution or the square root transformation with mirroring for left-skewed distribution); and (4) the transformed values were standardized with *z*-score using the mean and s.d.; these pre-processed 51 variables were used as the numeric physiological features (Supplementary Data 4). Likewise, the Arivale datasets of the obesity-related clinical blood markers (that is, selected clinical labs; Supplementary Data 6) and the TwinsUK datasets of the obesity-related phenotypic measures (Supplementary Data 6) were pre-processed. For gut microbiome α-diversity metrics, the number of observed ASVs and Chao1 index were converted with square root transformation, and Shannon’s index was converted with square transformation, and then these transformed values were standardized with *z*-score using the mean and s.d. Relationships of the numeric physiological features with the measured or omics-inferred BMI (Fig. 1e) were independently assessed using each OLS linear regression model with the (unstandardized) log_*e*_-transformed measured or omics-inferred BMI as a dependent variable, a feature as an independent variable and sex, age and ancestry principal components 1–5 as covariates while adjusting multiple testing across the 255 (51 features × 5 BMI types) regressions. Relationships between Measure (that is, BMI or WHtR) and the analytes that were retained in at least one of ten LASSO models (Fig. 2b–d and Extended Data Fig. 7k) were independently assessed using each OLS linear regression model with the (unstandardized) log_*e*_-transformed Measure as a dependent variable, an analyte as an independent variable and sex, age and ancestry principal component 1–5 as covariates while adjusting multiple testing across the 210 (Fig. 2b), 75 (Fig. 2c), 42 (Fig. 2d) or 289 (Extended Data Fig. 7k) regressions. In this regression analysis, a model including the omics-inferred Measure as an independent variable was also assessed as reference. Differences in ΔMeasure (that is, ΔBMI or ΔWHtR) between clinically defined metabolic health conditions (Fig. 3b and Extended Data Figs. 6a and 7m) were independently assessed using each OLS linear regression model with ΔMeasure as a dependent variable, metabolic condition (that is, healthy versus unhealthy) as a categorical independent variable and Measure, sex, age and ancestry principal components 1–5 as covariates while adjusting multiple testing across the eight (2 BMI classes × 4 omics categories; Fig. 3b and Extended Data Fig. 7m) or four (2 BMI classes × 2 cohorts; Extended Data Fig. 6a) regressions. Differences in the obesity-related clinical blood markers, the BMI-associated numeric physiological features or the gut microbiome α-diversity metrics between the misclassification strata against the omics-inferred BMI class (Figs. 3d,e and 4b and Extended Data Fig. 6c) were independently assessed using each OLS linear regression model with a marker, feature or metric as a dependent variable, misclassification (that is, matched versus mismatched) as a categorical independent variable and BMI, sex, age and ancestry principal components 1–5 as covariates while adjusting multiple testing across the 40 (2 BMI classes × 2 omics categories × 10 markers; Fig. 3d), 216 (2 BMI classes × 4 omics categories × 27 features; Fig. 3e), 24 (2 BMI classes × 4 omics categories × 3 metrics; Fig. 4b) or 24 (2 BMI classes × 12 measures; Extended Data Fig. 6c) regressions. In the above regression analyses for the TwinsUK cohort, ancestry principal components were eliminated from the covariates owing to data availability.

### Data visualization

Results were visualized using Python matplotlib (version 3.4.3) and seaborn (version 0.11.2) libraries, except for the plasma analyte correlation network. Data were summarized as the mean with 95% confidence interval or the standard box plot (median: center line; 95% confidence interval around median: notch; [*Q*_1_, *Q*_3_]: box limits; [*x*_min_, *x*_max_]: whiskers, where *Q*_1_ and *Q*_3_ are the 1st and 3rd quartile values, and *x*_min_ and *x*_max_ are the minimum and maximum values in [*Q*_1_ − 1.5 × IQR, *Q*_3_ + 1.5 × IQR] (IQR, interquartile range, *Q*_3_ − *Q*_1_), respectively), as indicated in each figure legend. For presentation purposes, confidence interval was simultaneously calculated during visualization using Python seaborn barplot or boxplot API with default setting (1,000 times bootstrapping or a Gaussian-based asymptotic approximation, respectively). The OLS linear regression line with 95% confidence interval was simultaneously generated during visualization using Python seaborn regplot API with default setting (1,000 times bootstrapping). The plasma analyte correlation network was visualized with a circos plot using the R circlize (version 0.4.15) package^60^.

### Reporting summary

Further information on research design is available in the Nature Portfolio Reporting Summary linked to this article.

## Online content

Any methods, additional references, Nature Portfolio reporting summaries, source data, extended data, supplementary information, acknowledgements, peer review information; details of author contributions and competing interests; and statements of data and code availability are available at 10.1038/s41591-023-02248-0.

## Supplementary information

Reporting Summary

Supplementary Data 1 Cohort summary. This .xlsx file contains demographic summary of the study cohorts and statistical test summaries for the independency of split sets. Descriptions about each sheet and each column are included in the README sheet.

Supplementary Data 2 Analytes of blood-measured omics. This .xlsx file contains information about the analytes of blood-measured omics and basic statistics of their baseline measurements. Descriptions about each sheet and each column are included in the README sheet.

Supplementary Data 3 *β*-coefficient estimates for the variables of the omics-based BMI models. This .xlsx file contains *β*-coefficient estimates for the variables of the omics-based BMI models, related to Fig. 2 and Extended Data Figs. 2–5 and 8. Descriptions about each sheet and each column are included in the README sheet.

Supplementary Data 4 Relationships of the numeric physiological measures with the measured or omics-inferred BMI. This .xlsx file contains the regression analysis summary for the association between each of the 51 numeric physiological measures and the measured or omics-inferred BMI, corresponding to Fig. 1e. Descriptions about each column are included in the README sheet.

Supplementary Data 5 Relationships of the retained analytes in the omics-based BMI models with BMI. This .xlsx file contains the regression analysis summary for the association between BMI and each of the analytes that were retained in at least one of ten LASSO models, corresponding to Fig. 2b–d. Descriptions about each sheet and each column are included in the README sheet.

Supplementary Data 6 Differences in phenotypic measures between the misclassification strata against the omics-inferred BMI class. This .xlsx file contains the regression analysis summary for the difference in the obesity-related clinical blood marker, the BMI-associated numeric physiological feature or the gut microbiome α-diversity metric between the misclassification strata against the omics-inferred BMI class, corresponding to Figs. 3d,e and 4b and Extended Data Fig. 6c. Descriptions about each sheet and each column are included in the README sheet.

Supplementary Data 7 Plasma analyte correlations modified by the baseline metabolic state and by lifestyle intervention. This .xlsx file contains the interaction analysis summary for the plasma analyte correlations modified by the baseline MetBMI and by days in program, corresponding to Fig. 6. Descriptions about each column are included in the README sheet.

Supplementary Data 8 *β*-coefficient estimates for the variables of the omics-based WHtR models. This .xlsx file contains *β*-coefficient estimates for the variables of the omics-based WHtR models, related to Extended Data Figs. 7 and 8. Descriptions about each sheet and each column are included in the README sheet.

Supplementary Data 9 Relationships of the retained analytes in the omics-based WHtR models with WHtR. This .xlsx file contains the regression analysis summary for the association between WHtR and each of the analytes that were retained in at least one of ten LASSO models, corresponding to Extended Data Fig. 7k. Descriptions about each sheet and each column are included in the README sheet.

Supplementary Data 10 Statistical test summary. This .xlsx file contains the statistical test summary, including sample size, degree of freedom, test statistic (nominal) *P* value and adjusted *P* value, corresponding to Figs. 1b–d, 3a,b and 4c–f and Extended Data Figs. 2d, 3d, 4a,b,f, 6a, 7c–e,l,m and 8d,e. Descriptions about each sheet and each column are included in the README sheet.

## Data Availability

The Arivale datasets that were used in this study are not publicly available owing to both ethical and legal reasons (see Reporting Summary), but qualified researchers can assess the de-identified datasets for research purposes through a Data Use Agreement. Inquiries about data access should be sent to data-access@isbscience.org and will be responded to within seven business days. The TwinsUK datasets that were used in this study were provided by the Department of Twin Research & Genetic Epidemiology (King’s College London) after the approval of our Data Access Application (project number E1192). The raw WMGS data of the TwinsUK cohort (without metadata) are publicly available on the NCBI Sequence Read Archive (https://www.ncbi.nlm.nih.gov/bioproject/PRJEB32731/). Requests should be referred to their website (http://twinsuk.ac.uk/resources-for-researchers/access-our-data/).

## Code availability

Code used in this study is freely available on GitHub (https://github.com/PriceLab/Multiomics-BMI).
