# Supplementary material for: Multiomic signatures of body mass index identify heterogeneous health phenotypes and responses to a lifestyle intervention
Source: Nat Med. 2023 Mar 20;29(4):996–1008. doi: 10.1038/s41591-023-02248-0 (PMC10115644; doi:10.1038/s41591-023-02248-0)
Supplement: Supplementary file 1 — Reporting Summary [file 41591_2023_2248_MOESM1_ESM.pdf]

nature portfolio

Corresponding author(s): Noa Rappaport

Last updated by author(s): Jan 23, 2023

Reporting Summary

Nature Portfolio wishes to improve the reproducibility of the work that we publish. This form provides structure for consistency and transparency in reporting. For further information on Nature Portfolio policies, see our Editorial Policies and the Editorial Policy Checklist.

Statistics

For all statistical analyses, confirm that the following items are present in the figure legend, table legend, main text, or Methods section.

n/a	Confirmed	
☐	☒	The exact sample size (n) for each experimental group/condition, given as a discrete number and unit of measurement
☐	☒	A statement on whether measurements were taken from distinct samples or whether the same sample was measured repeatedly
☐	☒	The statistical test(s) used AND whether they are one- or two-sided<br>*Only common tests should be described solely by name; describe more complex techniques in the Methods section.*
☐	☒	A description of all covariates tested
☐	☒	A description of any assumptions or corrections, such as tests of normality and adjustment for multiple comparisons
☐	☒	A full description of the statistical parameters including central tendency (e.g. means) or other basic estimates (e.g. regression coefficient) AND variation (e.g. standard deviation) or associated estimates of uncertainty (e.g. confidence intervals)
☐	☒	For null hypothesis testing, the test statistic (e.g. F, t, r) with confidence intervals, effect sizes, degrees of freedom and P value noted<br>*Give P values as exact values whenever suitable.*
☒	☐	For Bayesian analysis, information on the choice of priors and Markov chain Monte Carlo settings
☒	☐	For hierarchical and complex designs, identification of the appropriate level for tests and full reporting of outcomes
☐	☒	Estimates of effect sizes (e.g. Cohen's d, Pearson's r), indicating how they were calculated

Our web collection on statistics for biologists contains articles on many of the points above.

Software and code

Policy information about availability of computer code

Data collection	For the Arivale data, blood was sampled by trained phlebotomists at LabCorp (Laboratory Corporation of America Holdings, North Carolina, USA) or Quest (Quest Diagnostics, New Jersey, USA) service centers. Blood metabolomics, proteomics, and clinical labs were generated by Metabolon, Inc. (North Carolina, USA), Olink Proteomics (Uppsala, Sweden), and LabCorp or Quest in a Clinical Laboratory Improvement Amendments-certified lab, respectively. Saliva was sampled at home and measured by ZRT Laboratory (Oregon, USA). Daily physical activity measures were collected using wearable device and generated by its default algorithm (Fitbit, Inc., California, USA). Stool samples were collected by participants at home and measured by DNA Genotek, Inc. (Ottawa, Canada). The obtained FASTQ files were processed using the mbtools workflow (version 0.37.1; https://github.com/Gibbons-Lab/mbtools), and taxonomy assignment was performed using the SILVA ribosomal RNA gene database (version 132).<br>The TwinsUK data was provided by Department of Twin Research & Genetic Epidemiology (King's College London). The raw data of whole metagenomic shotgun sequencing was obtained from the National Center for Biotechnology Information (NCBI) Sequence Read Archive (PRJEB32731), and applied to a processing pipeline on Nextflow (version 22.04.5; https://github.com/Gibbons-Lab/pipelines). Through this pipeline, the obtained FASTQ files were processed using the fastp (version 0.23.2) tool (ref. 55) to filter and trim the reads, and taxonomic abundance was obtained using the Kraken 2 (version 2.1.2) and Bracken (version 2.6.0) tools (ref. 56) with the Kraken 2 default database (based on NCBI RefSeq).
Data analysis	Data was processed and analyzed using Python 3 (version 3.7.6 or 3.9.6) with Python NumPy (version 1.18.1 or 1.21.3) and pandas (version 1.0.3 or 1.3.4) libraries. The omics-based BMI and WHtR models and the gut microbiome-based obesity classifiers were generated using Python scikit-earn (version 1.0.1) library. LMMs, GLMs, and GEEs were modeled using Python statsmodels (version 0.13.0) library. Statistical analysis was performed using Python SciPy (version 1.4.1 or 1.7.1) and statsmodels (version 0.11.1 or 0.13.0) libraries and R pROC (version 1.18.0) package (ref. 58). Results were visualized using Python matplotlib (version 3.4.3) and seaborn (version 0.11.2) libraries and R circlize

(version 0.4.15) package (ref. 60). Code required to replicate the results of this study is freely available on GitHub (https://github.com/PriceLab/Multiomics-BMI).

For manuscripts utilizing custom algorithms or software that are central to the research but not yet described in published literature, software must be made available to editors and reviewers. We strongly encourage code deposition in a community repository (e.g. GitHub). See the Nature Portfolio guidelines for submitting code & software for further information.

Data

Policy information about availability of data

All manuscripts must include a data availability statement. This statement should provide the following information, where applicable:

- Accession codes, unique identifiers, or web links for publicly available datasets
- A description of any restrictions on data availability
- For clinical datasets or third party data, please ensure that the statement adheres to our policy

The Arivale datasets that were used in this study were originally generated by Arivale's commercial service. Institute for Systems Biology (ISB) and Arivale have an Asset License Agreement, which gives us the access to de-identified datasets from Arivale commercial subscribers. Because of ethical and legal points in the agreement, we are not permitted to upload the Arivale datasets to public databases. However, to facilitate collaborative validation and follow-up studies, ISB can share the Arivale de-identified datasets on the basis of signing a Data Use Agreement (DUA) that governs use of the data. The restrictions are consistent with general DUAs by other controlled-access databases (e.g., dbGaP): the recipient will not disclose the data to 3rd parties who themselves have not signed the DUA; the recipient will not attempt to re-identify the participants from their data; and the recipient may only use the data for non-commercial purposes. Inquiries about the data access should be sent to data-access@isbscience.org, and will be responded to within seven business days.
The TwinsUK datasets that were used in this study were provided by Department of Twin Research & Genetic Epidemiology (King's College London) after the approval of our Data Access Application (Project Number: E1192). The raw WMGS data of TwinsUK cohort (without metadata) is publicly available on the NCBI Sequence Read Archive (https://www.ncbi.nlm.nih.gov/bioproject/PRJEB32731/). Requests should be referred to their website (http://twinsuk.ac.uk/resources-for-researchers/access-our-data/).

Human research participants

Policy information about studies involving human research participants and Sex and Gender in Research.

Reporting on sex and gender	We followed the Sex and Gender Equity in Research (SAGER) guideline and included the recommended information in our manuscript. This study relied on self-reported sex in both Arivale and TwinsUK cohorts, and our findings apply to both sexes. This study was conducted with de-identified data of the participants who had consented to the use of their anonymized data in research. We did not have access to gender information in both cohorts. The number of individuals from each sex was clearly reported in Methods, and demographics was provided for each sex (Extended Data Fig. 1, Supplementary Data 1). In all the statistical analyses, we adjusted for sex. Moreover, we addressed sex-specific models for BMI (Extended Data Fig. 2d).
Population characteristics	A detailed description of population characteristics was provided for each sex (Extended Data Fig. 1, Supplementary Data 1, Methods).
Recruitment	We did not play a role in recruiting participants for the current study.<br>The Arivale participants were self-enrolled in the Arivale program, since it was a commercial subscription service. An individual was eligible for enrollment between 2015–2019 if the individual was over 18 years old, not pregnant, and a resident of any U.S. state except New York; participants were primarily recruited from Washington, California, and Oregon. The participants were not screened for any particular disease. Upon entering the program, the participants were provided with the option to permit the use of their de-identified data for scientific discovery. The participants who had consented to this option joined the research cohort, and were analylzed in the current study.<br>The TwinsUK participants voluntarily joined to the TwinsUK Registry, a British national register of adult twins (Ref. 31). The participants must be twins, and were recruited by media campaigns without screening for any particular disease. The participants had two or more clinical visits for biological sampling between 1992–2022. The de-identified data of the participants who had consented to the use of their anonymized data in research was provided by Department of Twin Research & Genetic Epidemiology (King's College London), and analyzed in the current study.
Ethics oversight	The current study was conducted with de-identified data of the participants who had consented to the use of their anonymized data in research. Procedures were run under the Western Institutional Review Board with Institutional Review Board (Study Number: 20170658 at Institute for Systems Biology and 1178906 at Arivale). Application of data access for the TwinsUK cohort was approved by the TwinsUK Resource Executive Committee (Project Number: E1192).

Note that full information on the approval of the study protocol must also be provided in the manuscript.

Field-specific reporting

Please select the one below that is the best fit for your research. If you are not sure, read the appropriate sections before making your selection.

☒ Life sciences ☐ Behavioural & social sciences ☐ Ecological, evolutionary & environmental sciences

For a reference copy of the document with all sections, see nature.com/documents/nr-reporting-summary-flat.pdf

Life sciences study design

All studies must disclose on these points even when the disclosure is negative.

Sample size	In both Arivale and TwinsUK datasets, sample size was not pre-determined by statistical methods, because the participant recruitment and data collections were done prior to the current study; i.e., the original datasets were collected independent from the aim of the current study. Instead, we used all the available datasets for the participants who satisfied the inclusion criteria (see next), and thus these inclusion criteria were the main factor for determining the sample size in the current study.
Data exclusions	In the current study, to compare the associations between BMI and host phenotypes across different omics, we limited our main study cohort to the Arivale participants whose datasets contained (1) all main omic measurements (metabolomics, proteomics, clinical laboratory tests) from the same first blood draw, (2) a BMI measurement within ±1.5 month from the first blood draw, and (3) genetic information (for using as covariates). Likewise, we limited the external cohort to the TwinsUK participants whose datasets contained all measurements for metabolomics, BMI, and the obesity-related standard clinical measures (i.e., triglycerides, HDL-cholesterol, LDL-cholesterol, glucose, insulin, and HOMA-IR) from the same visit. In addition, we eliminated (1) outlier participants whose baseline BMI was beyond ±3 s.d. from the mean in the baseline BMI distribution and (2) participants whose any of omic datasets contained more than 10% missingness in the filtered analytes. This elimination is because penalized regression is sensitive to outliers which skews the resulting models, and because imputation for too much missingness weights on available data which results in biased models. The final Arivale and TwinsUK cohorts consisted of 1,277 (821 female and 456 male) and 1,834 (1,774 female and 60 male) participants, respectively (Fig. 1a, Extended Data Fig. 1, Supplementary Data 1), which exhibited consistent demographics with the study cohorts defined in the previous studies (Ref. 20,25–29).<br>For the analyses of gut microbiome, the 702 (486 female and 216 male) and 329 (307 female and 22 male) participants were selected from the Arivale and TwinsUK cohorts, respectively, who collected a stool sample within ±1.5 month from the first blood draw and did not use antibiotics (Fig. 4a, Supplementary Data 1). This is because we needed to compare the gut microbiome profiles with the blood omic profiles, and because antibiotics directly affects the gut microbiome ecosystem.<br>For longitudinal analyses, the 608 (410 female and 198 male) participants were selected from the Arivale cohort, whose datasets contained two or more time-series datasets for both BMI and omics during 18 months after enrollment (Fig. 5a, Supplementary Data 1). This is because we cannot perform longitudinal analyses without data from more than two time points.<br>For the analyses of WHtR, the 1,078 (689 female and 389 male) participants were selected from the Arivale cohort, whose datasets contained the baseline WHtR measurement within ±1.5 month from the first blood draw and within ±3 s.d. from the mean in the baseline WHtR distribution (Extended Data Fig. 7a, Supplementary Data 1). This is because we needed to generate and compare WHtR models as well as BMI models.<br>In "Plasma analyte correlation network analysis" and "Statistical analysis", outlier values which were beyond ±3 s.d. from mean in the target cohort were eliminated. This is because the models is sensitive to outliers and their elimination allows convergence in modeling.<br><br>All these inclusions/exclusions criteria were also described in the Methods section.
Replication	Due to the nature of observational study, we cannot deny the possibility that the findings are restricted to our studied cohorts. However, to mitigate this limitation, we generated models while splitting dataset into training and testing datasets with a tenfold cross-validation scheme, and evaluated all the models with the hold-out testing set and confirmed robustness of parameters (e.g., Extended Data Fig. 2e–h). Moreover, we utilized the external TwinsUK cohort to validate the findings observed in the Arivale cohort.
Randomization	In both Arivale and TwinsUK datasets, the participant recruitment and data collections were done prior to the current study; i.e., the original datasets were collected independent from the aim of the current study. Hence, the longitudinal analyses in the Arivale cohort were unable to be designed as a randomized control trial in advance, and no randomization was performed in lifestyle intervention (i.e., all participants received lifestyle intervention). This was clearly described as a limitation in Discussion section. In data analysis, where appropriate, our statistical models were adjusted for covariates including sex, age, ancestry principal components, and meteorological seasons. The variables adjusted for each regression model were described in figure legend and Methods section.
Blinding	Because completely different researchers performed data collection and data analysis independently, further blinding was not performed in this study.

Reporting for specific materials, systems and methods

We require information from authors about some types of materials, experimental systems and methods used in many studies. Here, indicate whether each material, system or method listed is relevant to your study. If you are not sure if a list item applies to your research, read the appropriate section before selecting a response.

Materials & experimental systems

n/a	Involved in the study
☒	☐ Antibodies
☒	☐ Eukaryotic cell lines
☒	☐ Palaeontology and archaeology
☒	☐ Animals and other organisms
☒	☐ Clinical data
☒	☐ Dual use research of concern

Methods

n/a	Involved in the study
☒	☐ ChIP-seq
☒	☐ Flow cytometry
☒	☐ MRI-based neuroimaging
